# Chitosan Hydrogel Enriched with Propolis from Chihuahua, Mexico: Physicochemical Characterization and Exploratory In Vivo Evaluation in a Murine Second-Degree Burn Model

**DOI:** 10.3390/polym18182231

**Published:** 2026-09-13

**Authors:** Lydia Paulina Loya-Hernández, Manuel Román-Aguirre, Silvia Lorena Montes-Fonseca, Juan Antonio Arreguín-Cano, César Iván Romo-Sáenz, Carlos Arzate-Quintana, Daniela Muela-Campos, Nubia Ivette Amaya-Olivas, Guillermo Martínez-Mata, Juan Guillermo Ayala-Soto, Celia María Quiñonez-Flores

**Affiliations:** 1 Facultad de Odontología, Universidad Autónoma de Chihuahua, Circuito Universitario s/n, Campus I, Chihuahua 31110, Mexico; lploya@uach.mx (L.P.L.-H.); jcanon@uach.mx (J.A.A.-C.); dmuela@uach.mx (D.M.-C.); gmata@uach.mx (G.M.-M.); 2 Centro de Investigación en Materiales Avanzados (CIMAV), Complejo Industrial Chihuahua, Miguel de Cervantes 120, Chihuahua 31136, Mexico; manuel.roman@cimav.edu.mx; 3 Escuela de Medicina y Ciencias de la Salud, Tecnológico de Monterrey, Heroico Colegio Militar 4700, Col. Nombre de Dios, Chihuahua 31300, Mexico; silvialorena.montes@tec.mx; 4 Facultad de Medicina y Ciencias Biomédicas, Universidad Autónoma de Chihuahua, Circuito Universitario s/n, Campus II, Chihuahua 31125, Mexico; carzate@uach.mx; 5 Facultad de Ciencias Químicas, Universidad Autónoma de Chihuahua, Circuito Universitario s/n, Campus II, Chihuahua 31125, Mexico; namaya@uach.mx (N.I.A.-O.); jayala@uach.mx (J.G.A.-S.)

**Keywords:** chitosan hydrogel, propolis, wound dressing, wound healing, burns

## Abstract

**Background/Objectives**: Second-degree burns require strategies that address microbial contamination, oxidative stress, exudate, and tissue repair. This study aimed to develop and evaluate a marine-derived chitosan hydrogel incorporating ethanolic extract of propolis (EEP) from Chihuahua, Mexico, for second-degree burn management. **Methods**: EEP was characterized using parameters established in NOM-003-SAG/GAN-2017 and complementary biological assays. Chitosan hydrogels containing 1%, 3%, and 5% (*w*/*w*) EEP were evaluated for physicochemical properties, swelling, mass loss, flavonoid release, antioxidant activity, antimicrobial performance, and cell viability. The 1% EEP hydrogel was further evaluated in a murine second-degree burn model. **Results**: Chihuahua propolis exhibited high phenolic (27.42 ± 2.53%) and flavonoid (9.23 ± 0.314%) contents. EEP incorporation modified the chitosan matrix, providing high swelling capacity, increased structural persistence, sustained flavonoid release for 72 h, and radical-scavenging activity for 96 h. Antimicrobial activity was concentration-dependent, with the 5% EEP hydrogel reducing recoverable *Escherichia coli* counts below the detection limit (<10^2^ CFU/mL). However, EEP-containing hydrogels reduced NIH-3T3 viability below the 70% ISO 10993-5 threshold under static extraction conditions. At 144 h, the 1% EEP hydrogel produced greater wound contraction than silver sulfadiazine (*p* = 0.021), although it did not differ significantly from the propolis-free chitosan hydrogel or untreated control. Qualitative histological assessment showed features consistent with early tissue repair, with cutaneous appendages observed in several sections from the 1% EEP group. **Conclusions**: Chihuahua propolis-loaded chitosan hydrogels showed promising physico-chemical, release, antioxidant, antimicrobial, and short-term in vivo findings. The in vitro reduction in metabolic activity observed under static extraction conditions warrants further evaluation using physiologically relevant exposure models, extended follow-up, and comprehensive safety assessment.

## 1. Introduction

The skin is the largest organ of the human body and serves as the primary line of defense against external threats, fulfilling critical roles in thermoregulation, immunological surveillance, and the maintenance of physiological homeostasis [1]. Its structural integrity acts as a selectively impermeable barrier against pathogens, mechanical insults, and chemical agents [2]. When this barrier is compromised, as occurs with burns, surgical incisions, or chronic ulcers, a precisely orchestrated cascade of cellular and molecular events is initiated to restore tissue continuity. This process, known as wound healing, proceeds through four overlapping phases: hemostasis, inflammation, proliferation, and tissue remodeling [3]. Any disruption to these stages, whether due to infection, excessive oxidative stress, or metabolic dysfunction can result in delayed healing, chronic wound formation, or pathological scarring, such as hypertrophic cicatrices [4,5].

Burn injuries constitute a significant global public health concern, particularly in low-income countries where access to specialized wound care is often limited [6]. The economic and psychosocial impact of burn injuries is substantial; treatment costs are considerable, and survivors frequently face long-term physical and psychological sequelae [7,8]. Second-degree burns, which extend through the epidermis into the dermis [9,10], are of particular clinical relevance due to their prevalence and the complexity of their management, which requires dressings capable of addressing multiple biological targets simultaneously [10]. Among the principal obstacles to burn wound recovery is microbial colonization. The exposed tissue of thermal injuries provides an ideal niche for bacterial proliferation [11]. Concurrently, the inflammatory milieu of burn wounds generates an excess of reactive oxygen species (ROS), which damage cellular lipids, proteins, and DNA, ultimately arresting cell cycle progression and perpetuating a pro-inflammatory state that impairs tissue regeneration [12].

Conventional topical treatments, including silver-based formulations and antibiotics, have long represented the standard of care for burn wound management owing to their broad-spectrum antimicrobial activity; however, prolonged use may be associated with cytotoxic effects, delayed epithelialization, and an increased risk of antimicrobial resistance [13,14]. These limitations underscore the need for multifunctional therapeutic systems that can simultaneously integrate antioxidant and antimicrobial activity with adequate biocompatibility and tissue compatibility. In this context, the development of bioactive wound dressings that can simultaneously address antimicrobial resistance, oxidative damage, and impaired tissue repair represents a priority in pharmaceutical research. Among natural products, propolis, a resinous substance collected by honeybees (*Apis mellifera*) from plant exudates, has attracted considerable scientific interest. Its complex composition, comprising numerous chemical constituents, including phenolic compounds and flavonoids, is associated with a broad range of reported biological activities, including antibacterial, antifungal, anti-inflammatory, and antioxidant effects [15,16]. The composition and biological activity of propolis can vary according to geographic origin, botanical sources, environmental conditions, and processing methods [17,18]. In Mexico, the quality of propolis intended for therapeutic use is addressed by the Norma Oficial Mexicana NOM-003-SAG/GAN-2017 [19], which establishes physical, chemical, and antimicrobial specifications and corresponding testing methods. Characterization according to this standard therefore provides a standardized basis for describing the quality-related properties of the propolis used in a formulation.

Hydrogels have emerged as highly versatile platforms for wound management, consisting of three-dimensional hydrophilic polymeric networks that can provide a moist environment and support the incorporation and local delivery of bioactive compounds. Their high water absorption capacity, ability to manage wound exudate, and compatibility with bioactive cargo make them particularly suitable for topical delivery applications [20]. Among hydrogel-forming polymers, chitosan, a marine-derived polysaccharide commonly obtained from the deacetylation of chitin recovered from crustacean processing by-products, has attracted considerable attention because of its biocompatibility, biodegradability, bioadhesive properties, and intrinsic antimicrobial activity. Moreover, its ability to promote hemostasis and serve as a carrier for bioactive compounds makes it an attractive material for advanced wound dressing and topical delivery applications [21,22]. The incorporation of natural bioactive compounds such as propolis into chitosan-based hydrogel matrices therefore represents a promising strategy for developing multifunctional topical systems that combine the properties of a marine-derived polymeric platform with the biological activities of propolis.

Chitosan–propolis materials have previously been investigated for wound-related applications. Therefore, the originality of the present study does not reside in the chitosan–propolis combination itself, but in the evaluation of an ethanolic extract of propolis from a geographically defined source in Chihuahua, Mexico, incorporated into a lyophilized chitosan hydrogel and examined through an integrated physicochemical, functional, and exploratory in vivo approach. To the best of our knowledge, this specific combination of Chihuahua propolis and a chitosan hydrogel has not previously been evaluated in a murine second-degree burn model. The geographical provenance of propolis is scientifically relevant because its composition may vary with geographic origin, botanical sources, environmental conditions, and processing; however, the present study does not establish a unique phytochemical profile for Chihuahua propolis. The resulting formulations were evaluated in terms of their physicochemical, release, antioxidant, antimicrobial, and cell viability properties, and the 1% EEP formulation was further assessed in a murine model of second-degree burns through macroscopic and descriptive histological evaluation.

## 2. Materials and Methods

### 2.1. Sample Collection

Raw propolis was collected from hives of the honeybee Apis mellifera at “El Girasol” apiaries, located northeast of the city of Cuauhtémoc, Chihuahua, Mexico (28°23′28″ N, 107°08′19″ W), at an altitude of 2184 m above sea level, during September 2024 using the scraping method. The collection period is reported because seasonal conditions may contribute to variability in propolis composition. The collected material was subsequently cleaned manually to remove visible impurities prior to further processing. The crude propolis was then characterized based on macroscopic and organoleptic parameters, including color, aroma, taste, and consistency.

### 2.2. Physicochemical Characterization of Propolis Extract

#### 2.2.1. Quality Parameters According to NOM-003-SAG/GAN-2017

The ethanolic extraction of propolis (EEP) and the quality-related analyses included in this section were performed in accordance with the specifications established by the Norma Oficial Mexicana NOM-003-SAG/GAN-2017 [19], which defines physical, chemical, and antimicrobial specifications and corresponding testing methods for propolis. The qualitative tests specified by the standard for the detection of phenolic and flavonoid compounds were also performed as preliminary screening and yielded positive results. Parameters evaluated quantitatively included total phenolic content, total flavonoid content, oxidation index, and antioxidant activity as applicable to the standard. Complementary biological assays were conducted separately to further characterize the EEP.

#### 2.2.2. Additional Physicochemical Characterization

Complementary analyses were performed to further characterize the biological safety and antimicrobial profile of the propolis ethanolic extract beyond the parameters required by NOM-003-SAG/GAN-2017.

##### Minimum Inhibitory Concentration

The minimum inhibitory concentration (MIC) of the EEP was determined by broth microdilution assay in 96-well microplates against *Candida albicans* (ATCC 14053), *Escherichia coli* O157:H7 (ATCC 43895), *Staphylococcus epidermidis* (ATCC 12228), and *Staphylococcus aureus* (ATCC 25923). Brain Heart Infusion (BHI) broth was used as the culture medium. A stock solution was prepared by dissolving propolis (250 mg) in 10% (*v*/*v*) dimethyl sulfoxide (DMSO) and sterilized by filtration through a 0.22 µm membrane filter. A two-fold dilution was performed to obtain a working solution of 125 mg/mL containing 5% (*v*/*v*) DMSO. For the assay, 50 µL of the extract was added to each well of a 96-well microplate, followed by 50 µL of sterile BHI broth and 50 µL of microbial inoculum adjusted to a final concentration of 1 × 10^6^ CFU/mL, resulting in a final volume of 150 µL per well. This corresponded to an initial extract concentration of 41.7 mg/mL and 1.67% (*v*/*v*) DMSO. Nine two-fold serial dilutions were prepared in the microplate, yielding final concentrations ranging from 41.7 to 0.16 mg/mL. Plates were incubated at 37 °C for 24 h. Wells containing only culture medium served as sterility controls, while inoculated broth without extract served as the positive growth control. Following incubation, 10 µL of resazurin solution was added to each well, and plates were further incubated for 2 h at 37 °C in the dark. The MIC was defined as the lowest extract concentration that prevented the color change in resazurin from blue to pink, indicative of microbial metabolic activity. Each assay was performed in triplicate, and the reported MIC value corresponded to the concentration consistently observed in at least two independent replicates.

##### *Artemia salina* Lethality Assay

*Artemia salina* cysts were hatched in aerated saline water (33 g/L NaCl) at 25–28 °C under constant illumination. After 48 h, ten actively swimming nauplii were transferred into individual vials containing 10 mL of freshly prepared saline solution. EEP solutions were prepared from a stock solution diluted in DMSO to a final solvent concentration of 1% (*v*/*v*) and tested at concentrations ranging from 1 to 500 µg/mL. Experimental vials were incubated at 25–28 °C for 24 h under illumination, after which nauplii survival was recorded. Individuals that showed no movement after gentle agitation of the vial were considered dead. Two controls were included: saline solution alone (negative control) and saline solution containing 1% (*v*/*v*) DMSO (solvent control). Each concentration was tested in triplicate. Acute toxicity was estimated as the median lethal concentration (LC_50_) by Probit analysis, with mortality data plotted against log_10_-transformed concentrations.

##### Hemolysis Assay

The hemolytic activity of the EEP was evaluated using human erythrocytes obtained from healthy volunteers. Blood samples were collected in EDTA-containing tubes and centrifuged at 1500× *g* for 10 min to remove plasma and the buffy coat. The erythrocyte pellet was washed four times with phosphate-buffered saline (PBS, pH 7.4) and resuspended in PBS to obtain a 5% (*v*/*v*) suspension. EEP was prepared at a concentration of 0.05 mg/mL and incubated with the erythrocyte suspension at 37 °C for 1 h under gentle agitation. PBS alone, 1% (*v*/*v*) Triton X-100, and 3.5% (*v*/*v*) ethanol were used as the negative control, positive control, and vehicle control, respectively. After incubation, samples were centrifuged at 1500× *g* for 10 min, and the absorbance of the supernatant was measured at 540 nm using a spectrophotometer. Each condition was tested in triplicate. The percentage of hemolysis was calculated using the following formula:

Hemolysis (%) = [(A sample − A negative control)/(A positive control − A negative control)] × 100

where A sample is the absorbance of erythrocytes incubated with the test sample, A negative control is the absorbance of erythrocytes incubated in PBS, representing 0% hemolysis, and A positive control is the absorbance of erythrocytes treated with TritonX-100 (representing 100% hemolysis).

##### Cell Viability Assessment in Fibroblasts

Murine fibroblasts NIH-3T3 (ATCC CRL-1658) were used to evaluate cell viability/metabolic activity after exposure to EEP. Cells were cultured in Dulbecco’s Modified Eagle’s Medium (DMEM, high glucose) supplemented with 10% (*v*/*v*) fetal bovine serum (FBS) and 1% (*v*/*v*) penicillin–streptomycin at 37 °C in a humidified atmosphere containing 5% CO_2_, and used between passages 5 and 10. EEP stock solutions were prepared in DMSO and subsequently diluted in phosphate-buffered saline (PBS, pH 7.4). Final concentrations of 200, 100, 50, 25, 10, 5, and 2.5 µg/mL were obtained by serial dilution in supplemented DMEM. Cells were seeded in 96-well plates at a density of 2.5 × 10^4^ cells per well in 50 µL of cell suspension, followed by the addition of 150 µL of medium containing the corresponding EEP concentration, resulting in a final volume of 200 µL per well. Each concentration was tested in six technical replicates. The final DMSO concentration did not exceed 0.2% (*v*/*v*) in any experimental condition. Untreated cells served as the viability control, while wells treated with DMSO at the maximum tested concentration were included as the solvent control. After 24 h of incubation, cell viability was assessed using a resazurin-based metabolic assay. Briefly, 10 µL of resazurin solution (0.015% *w*/*v*) was added to each well and incubated for 4 h at 37 °C. Fluorescence was measured using a Varioskan™ LUX multimode microplate reader (Thermo Scientific, Waltham, MA, USA) at excitation and emission wavelengths of 560 nm and 590 nm, respectively. Results were expressed as the percentage of cell viability relative to untreated controls, calculated as follows:

Cell viability (%) = (F treated/F untreated control) × 100

where F treated is the fluorescence intensity of NIH-3T3 fibroblasts exposed to the corresponding EEP concentration, and F untreated control is the fluorescence intensity of untreated cells cultured under identical conditions, representing 100% cell viability.

### 2.3. Preparation of Chitosan–Propolis Hydrogels

Chitosan (degree of deacetylation ≥ 75%; Sigma-Aldrich, St. Louis, MO, USA) was used as the polymeric matrix for hydrogel preparation. Briefly, 2 g of chitosan was dissolved in 100 mL of 2% (*v*/*v*) acetic acid under continuous magnetic stirring at 60 °C for 24 h until complete solubilization. Glycerol was subsequently incorporated as a plasticizer at a ratio of 1 mL per gram of chitosan, and the mixture was further homogenized. The pH was then adjusted to 6.5 by dropwise addition of 0.5 M NaOH under constant stirring. Following neutralization, the mixture was maintained under stirring at room temperature for an additional 24 h to ensure homogeneity. The EEP was incorporated into the chitosan solution based on weight-to-weight (*w*/*w*) calculations relative to the initial chitosan mass, to obtain final formulations containing 1%, 3%, and 5% (*w*/*w*) EEP. A propolis-free control formulation was prepared under identical conditions. The resulting dispersions were poured into circular Teflon molds, pre-frozen at −60 °C for 3 h, and subsequently lyophilized for 72 h using a Base freeze-dryer to obtain the chitosan–propolis hydrogels. The resulting hydrogels were stored under dry conditions at room temperature until further characterization.

### 2.4. In Vitro Release of Flavonoids from Propolis-Loaded Chitosan Hydrogels

The release of flavonoids from lyophilized chitosan hydrogels containing 1%, 3%, and 5% (*w*/*w*) EEP was evaluated using a total immersion method. Hydrogel samples of approximately 1 cm^2^ were immersed in 20 mL of phosphate-buffered saline (PBS, pH 7.4) and incubated at 37 °C under constant agitation. At predetermined time intervals ranging from 2.5 to 72 h, 1 mL aliquots of the release medium were withdrawn and immediately replaced with an equal volume of fresh PBS to maintain sink conditions and a constant total volume throughout the experiment. The withdrawn samples were analyzed for flavonoid concentration by UV-Vis spectrophotometry at 415 nm, and results were expressed as quercetin equivalents (QE) using a previously established calibration curve. To account for potential interference from the polymeric matrix, absorbance values were corrected using propolis-free chitosan hydrogels as blank controls. All experiments were performed in triplicate. This assay was designed to compare the release behavior of the formulations under controlled in vitro conditions:

n − 1

Mt = Ct × V + Σ Ci × Vs

i = 1

where Mt represents the cumulative amount of flavonoids released at time t, Ct is the flavonoid concentration in the release medium at time t, V is the total volume of the release medium (20 mL), Ci is the flavonoid concentration measured at each previous sampling point, Vs corresponds to the volume of each withdrawn aliquot (1 mL), and n − 1 denotes the number of sampling points preceding time t, excluding the current measurement to avoid double-counting with the first term of the equation.

### 2.5. Swelling Behavior of Propolis-Loaded Chitosan Hydrogels

The swelling behavior of the lyophilized chitosan–propolis hydrogels was evaluated under physiological conditions. Lyophilized samples were cut into specimens with a surface area of approximately 1 cm^2^ and uniform thickness. The initial dry weight of each specimen (W_0_) was recorded prior to immersion. Each hydrogel specimen was individually immersed in phosphate-buffered saline (PBS, pH 7.4) and incubated at 37 °C under constant agitation. At predetermined time intervals (30 min, 1, 2, 4, 8, 24, and 48 h), samples were removed from the medium, gently blotted with filter paper to remove surface-adhered PBS, and immediately weighed to determine the swollen weight (Wt). Following each measurement, samples were returned to fresh PBS and incubated under the same conditions. The swelling percentage (SP) at each time point was calculated using the following equation:

SP (%) = [(Wt − Wo)/Wo)] × 100

where Wo represents the initial dry weight of the hydrogel specimen and Wt corresponds to the weight of the swollen sample at time t. All experiments were performed in triplicate for each formulation.

### 2.6. In Vitro Degradation of Propolis-Loaded Chitosan Hydrogels

The in vitro degradation of the chitosan–propolis hydrogels was evaluated by mass loss analysis. The same specimens used in the swelling assay were employed for this study, following the final swelling measurement at 48 h. After completion of the swelling assay, the hydrogel specimens remained immersed in phosphate-buffered saline (PBS, pH 7.4) at 37 °C under constant agitation for an additional period of 14 days. The PBS medium was renewed every 24 h throughout the incubation period to maintain stable physicochemical conditions. At the end of the degradation period, specimens were removed from the medium and dried at room temperature until constant weight was reached. The final dry weight of each specimen (Wf) was then recorded. The percentage of mass loss (degradation) was calculated using the following equation:

Degradation (%) = [(Wo − Wf)/Wo)] × 100

where Wo represents the initial dry weight of the hydrogel specimen prior to the swelling assay and Wf corresponds to the final dry weight recorded after the 14-day degradation period. All experiments were performed in triplicate for each formulation.

### 2.7. Scanning Electron Microscopy Analysis

The surface and cross-sectional morphology of the lyophilized chitosan–propolis hydrogels were examined by scanning electron microscopy (SEM) using a Hitachi SU3500 microscope (Hitachi High-Tech, Tokyo, Japan). Both the propolis-free control hydrogel and hydrogels containing 1%, 3%, and 5% (*w*/*w*) EEP were analyzed. To expose the internal micro-structure, specimens were fractured cryogenically by immersion in liquid nitrogen prior to cutting, yielding clean cross-sectional surfaces. The fractured specimens were mounted on aluminum stubs using conductive carbon tape. Prior to imaging, all specimens were sputter-coated with a thin gold layer to enhance electrical conductivity and image quality. Micrographs were acquired at various magnifications to evaluate pore morphology, size distribution, and overall structural characteristics of the hydrogels.

### 2.8. Thermogravimetric Analysis and Differential Scanning Calorimetry

The thermal properties of the lyophilized chitosan–propolis hydrogels were characterized by simultaneous thermo-gravimetric analysis (TGA) and differential scanning calorimetry (DSC) using an SDT Q600 analyzer (TA Instruments Inc., New Castle, DE, USA). Samples were placed in aluminum crucibles and heated from room temperature to 800 °C at a heating rate of 10 °C/min under an air atmosphere. This thermal program enabled the simultaneous assessment of mass loss, thermal stability, degradation stages, and thermal transitions associated with the individual components of the material.

### 2.9. Fourier Transform Infrared Spectroscopy

The chemical structure and functional groups of the propolis-free chitosan hydrogel and the chitosan–propolis hydrogels containing 1%, 3%, and 5% (*w*/*w*) EEP were analyzed by Fourier transform infrared spectroscopy in attenuated total reflectance mode (FTIR-ATR). Measurements were performed using a Shimadzu IRAffinity-1S spectrometer (Shimadzu Corporation, Kyoto, Japan) equipped with a diamond ATR crystal (2 mm diameter). Spectra were collected over a wavenumber range of 4000–450 cm^−1^ at a resolution of 4 cm^−1^, and each spectrum was obtained by co-averaging 40 scans.

### 2.10. Antioxidant Activity of Compounds Released from Hydrogels

The antioxidant capacity of the compounds released from the chitosan–propolis hydrogels was determined using the DPPH radical scavenging assay. Lyophilized hydrogel specimens were cut into pieces of approximately 1 cm^2^, following the same procedure described in previous experiments. Each specimen was immersed in phosphate-buffered saline (PBS, pH 7.4) and incubated at 37 °C under continuous agitation to allow the release of bioactive compounds. At predetermined time intervals (1, 2.5, 8.5, 24, 48, 72, and 96 h), aliquots of the release medium were collected and immediately replaced with an equal volume of fresh PBS to maintain a constant system volume. For the DPPH assay, 100 µL of the collected release medium was mixed with 100 µL of DPPH solution, resulting in a 1:1 (*v*/*v*) reaction mixture. The mixture was incubated in the dark at room temperature for 30 min. Absorbance was subsequently measured at 540 nm using a UV-Vis spectrophotometer. The percentage of DPPH radical inhibition was calculated using the following equation:

DPPH inhibition (%) = [(A control − A sample)/(A control)] × 100

where A control corresponds to the absorbance of the DPPH solution without sample and A sample corresponds to the absorbance of the reaction mixture at each time point. All measurements were carried out in triplicate.

### 2.11. Antimicrobial Activity of Propolis-Loaded Hydrogels

The antimicrobial activity of the chitosan–propolis hydrogels was assessed using two complementary methods: the agar disc diffusion assay and the drop plate method for quantification of viable microorganisms. For the agar disc diffusion assay, *Candida albicans* (ATCC 14053), *Escherichia coli* O157:H7 (ATCC 43895), *Staphylococcus epidermidis* (ATCC 12228), and *Staphylococcus aureus* (ATCC 25923) were adjusted to a turbidity equivalent to the 0.5 McFarland standard. Suspensions were uniformly spread onto Mueller–Hinton agar plates using the lawn culture technique. Lyophilized hydrogel specimens were sterilized by exposure to ultraviolet (UV) radiation for 30 min on each side and cut into discs with a diameter comparable to that of commercial antibiotic susceptibility discs. The discs were carefully placed onto the surface of the inoculated agar plates. Fluconazole discs served as the positive control for *C. albicans*, amoxicillin discs for *S. aureus* (ATCC 25923) and *S. epidermidis* (ATCC 12228), and ciprofloxacin discs for *E. coli* O157:H7 (ATCC 43895). Ethanol-impregnated discs were included as negative controls. Plates were incubated aerobically at 37 °C for 24 h. Antimicrobial activity was evaluated by measuring the diameter of inhibition zones (mm) formed around each hydrogel disc. All experiments were performed in triplicate.

For the drop plate method, lyophilized hydrogel specimens of approximately 1 cm^2^ were sterilized by UV irradiation for 30 min and inoculated with 100 µL of microbial suspension adjusted to 0.5 McFarland standard for each strain. After incubation at room temperature for 24 h, microorganisms adhered to the hydrogel surface were recovered by washing each specimen with 10 mL of sterile 0.1% (*w*/*v*) peptone water. Serial decimal dilutions were prepared, and 10 µL aliquots were plated onto nutrient agar using the drop plate technique. Plates were incubated at 37 °C for 24 h, and colony-forming units (CFU) were enumerated. The microbial load was calculated as CFU/mL using counts from dilutions yielding 3–30 colonies per drop. Results were expressed as log_10_ CFU/mL. When no colonies were detected, values were reported as below the detection limit (<10^2^ CFU/mL).

### 2.12. Cell Viability Evaluation of Hydrogels by Extract Method

Murine fibroblasts NIH-3T3 (ATCC CRL-1658) were used to evaluate cell viability/metabolic activity after exposure to conditioned media generated from the chitosan–propolis hydrogels under the same culture conditions previously described for the EEP assay. Hydrogel specimens (propolis-free chitosan hydrogel and propolis-loaded formulations containing 1%, 3%, and 5% *w*/*w* EEP) were cut into pieces of approximately 1 cm^2^ (≈30 mg per specimen) and sterilized by UV irradiation for 30 min on each side. Each hydrogel specimen was then immersed in complete culture medium and incubated for 24 h under standard culture conditions to obtain the material-conditioned medium. NIH-3T3 cells were seeded in 96-well plates at a density of 2.5 × 10^4^ cells per well in 50 µL of cell suspension. Subsequently, 150 µL of hydrogel-conditioned medium, previously diluted to the desired concentrations (100%, 50%, 25%, and 10%) in complete culture medium, was added to each well, resulting in a final volume of 200 µL. Untreated cells cultured in complete medium served as the viability control. Cell viability was assessed after 24 h of incubation using the resazurin-based metabolic assay as described above, and results were expressed as the percentage of cell viability relative to untreated controls.

### 2.13. Murine Burn Model

#### 2.13.1. Experimental Animals

Eight-week-old male BALB/c mice were obtained from the Animal Facility of the Faculty of Medicine and Biomedical Sciences, Autonomous University of Chihuahua (Chihuahua, México). Animals were housed under controlled environmental conditions (23 ± 1 °C, 36 ± 1% relative humidity, and a 12 h light/dark cycle) with ad libitum access to food and water. All experimental procedures were conducted in strict accordance with the Mexican Official Standard NOM-062-ZOO-1999 [23] for the care and use of laboratory animals and were approved by the Institutional Research Committee of the Autonomous University of Chihuahua (protocol CI-003-25).

#### 2.13.2. Experimental Groups and Treatments

Mice were randomly assigned to four experimental groups (n = 6 per group): (1) untreated control, (2) positive control treated with commercial silver sulfadiazine ointment, (3) propolis-free chitosan hydrogel, and (4) chitosan hydrogel containing 1% (*w*/*w*) EEP. The initial sample-size requirement was determined a priori using the resource equation approach because a reliable effect size could not be assumed for this exploratory animal experiment. With four experimental groups and five animals per group, the error degrees of freedom were E = N − k = 20 − 4 = 16, within the commonly recommended range of approximately 10–20 for this approach [24]. One additional animal was included per group to compensate for potential losses, resulting in six animals per group (24 animals in total; E = 24 − 4 = 20). The four groups were selected to distinguish the early response of untreated wounds, a clinically established reference treatment, the contribution of the chitosan matrix, and the selected 1% EEP-containing formulation. Silver sulfadiazine ointment was applied every 24 h, while hydrogel dressings of approximately 1 × 1 cm were placed directly over the burn area and replaced every 48 h. All treatments were administered for 6 days following burn induction. General clinical signs, including coat condition, posture, and spontaneous activity, were monitored daily throughout the experimental period.

#### 2.13.3. Burn Induction Procedure

Mice were anesthetized by inhalation of sevoflurane in an induction chamber until a deep anesthetic plane was achieved; anesthesia was maintained via facemask, when necessary, throughout the procedure. Under deep anesthesia, the dorsal area was shaved using an electric clipper, followed by the application of a thin layer of commercial depilatory cream for 2 min, which was subsequently removed with a moistened cotton swab by gentle rubbing against the direction of hair growth. A partial-thickness second-degree burn was then induced on the depilated dorsal region using a preheated aluminum block. The aluminum block was preheated until its surface reached 120 °C and then applied to the dorsal skin surface for 10 s to generate a controlled and reproducible burn injury. Following the procedure, animals were placed in a thermally controlled recovery area to prevent hypothermia. Analgesia was provided by administering paracetamol (200 mg/kg body weight) dissolved in the drinking water, and mice were housed individually during the recovery period.

#### 2.13.4. Wound Closure

Wound healing was monitored at 96 and 144 h post-burn induction using a digital caliper to record the length and width of the lesion. Given the approximately rectangular shape of the burn wound, the area was estimated from these measurements. The percentage of wound contraction was calculated relative to the initial wound area recorded on day 0, according to the following formula:

Wound contraction (%) = [(A0 − At)/A0] × 100

where A0 represents the initial wound area and At corresponds to the wound area at each measurement time point. This allowed quantitative assessment of progressive wound closure throughout the experimental period.

#### 2.13.5. Histological Analysis

At the end of the experimental period, tissue samples from the burn area were collected from all animals following euthanasia. Specimens were fixed in 10% neutral buffered formalin for 24 h, processed through graded ethanol series, embedded in paraffin, and sectioned at 5 µm thickness. Histological evaluation was performed as an exploratory descriptive assessment of tissue repair. Hematoxylin and eosin (H&E)-stained sections from all animals were examined independently by two pathologists blinded to the treatment groups. The evaluation focused on identifying the predominant histopathological features associated with wound healing, including epithelial continuity, inflammatory infiltrate, granulation tissue formation, collagen deposition and organization, angiogenesis, residual necrotic tissue, and the presence of cutaneous appendages. Following independent examination, the pathologists reached a consensus descriptive interpretation for each experimental group based on the overall tissue characteristics observed across the analyzed sections. Given the exploratory nature of the study, no histological scoring system, semiquantitative grading scale, or morphometric analysis was applied. Therefore, histological findings are presented as a qualitative descriptive assessment intended to support the macroscopic and biological observations, and no inferential statistical analysis was performed for histological outcomes.

## 3. Results

### 3.1. Physicochemical and Biological Characterization of Chihuahua Propolis and Its Ethanolic Extract

**Figure 1:**
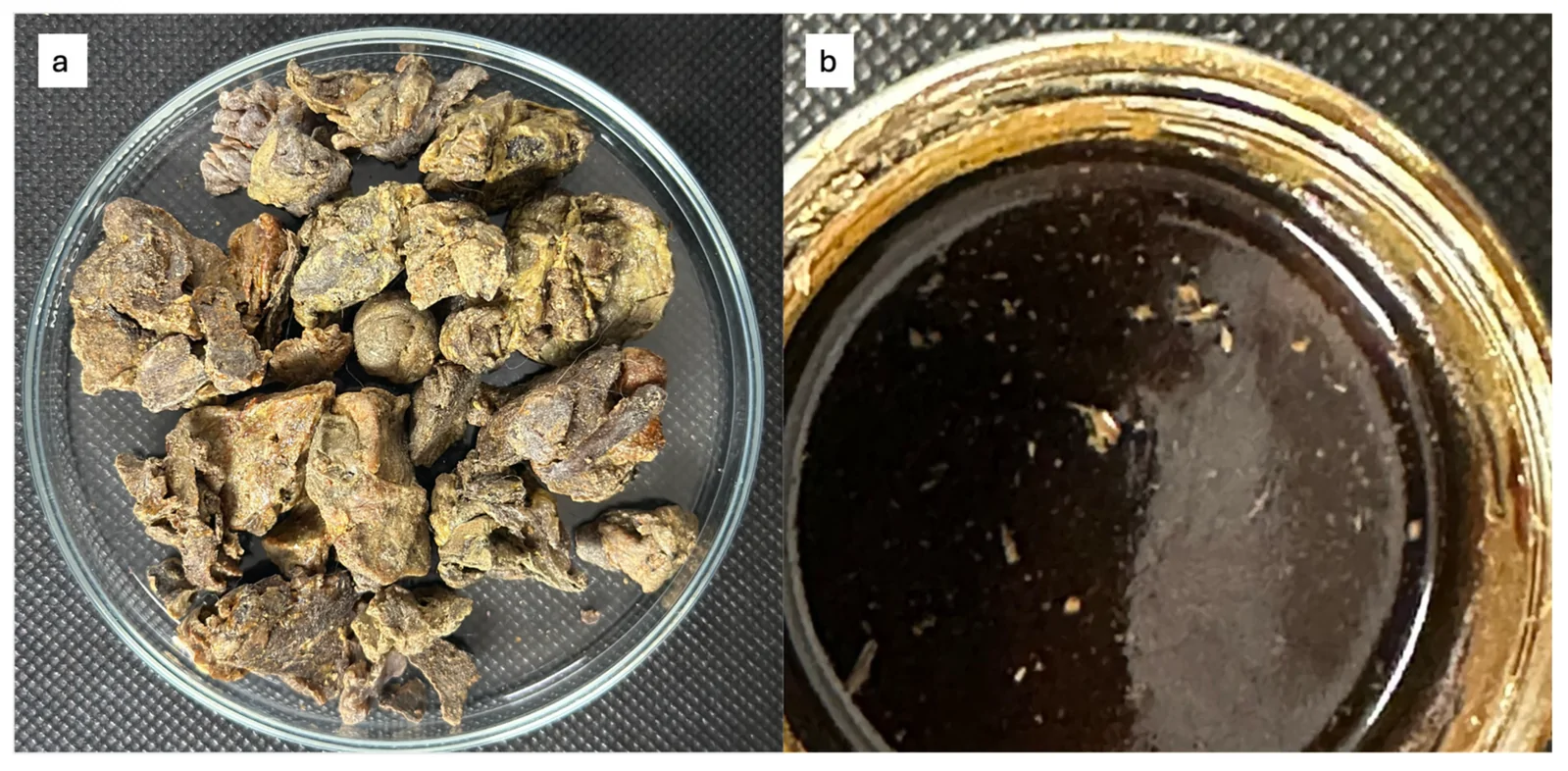
(**a**) Raw propolis freshly collected from the beehive. (**b**) Ethanolic extract of propolis.

Raw propolis collected from the beehives presented as irregular solid fragments with a greenish-brown coloration, a pungent balsamic aroma, a pungent taste, and malleable consistency (Figure 1a). The EEP obtained from this material exhibited a deep dark brown coloration (Figure 1b). The physicochemical and biological parameters evaluated are summarized in Table 1. The antioxidant activity of the EEP yielded an IC_50_ value of 184.00 ± 9.70 µg/mL. The oxidation index was 31.33 ± 6.11 s, exceeding the minimum threshold of 22 s established by NOM-003-SAG/GAN-2017. Total phenolic content was 27.42 ± 2.53% gallic acid equivalents and total flavonoid content was 9.23 ± 0.314% quercetin equivalents. Hemolytic activity against human erythrocytes was 0.006 ± 0.0039%, indicating negligible erythrocyte lysis at the tested concentration. The brine shrimp lethality assay yielded an LC_50_ value of 26.40 ± 4.75 µg/mL. Antimicrobial activity assessed by the agar disc diffusion assay demonstrated inhibition zones of 21.66 ± 4.16 mm, 14.33 ± 0.57 mm, and 12.66 ± 0.57 mm against *C. albicans*, *S. aureus*, and *S. epidermidis*, respectively. No inhibition zone was detected against *E. coli* O157:H7. Minimum Inhibitory Concentration (MIC) values were 2.60 mg/mL for *S. aureus* and *C. albicans*, 5.20 mg/mL for *S. epidermidis*, and 10.41 mg/mL for *E. coli* O157:H7.

#### 3.1.1. Cell Viability Assessment in Fibroblasts

**Figure 2:**
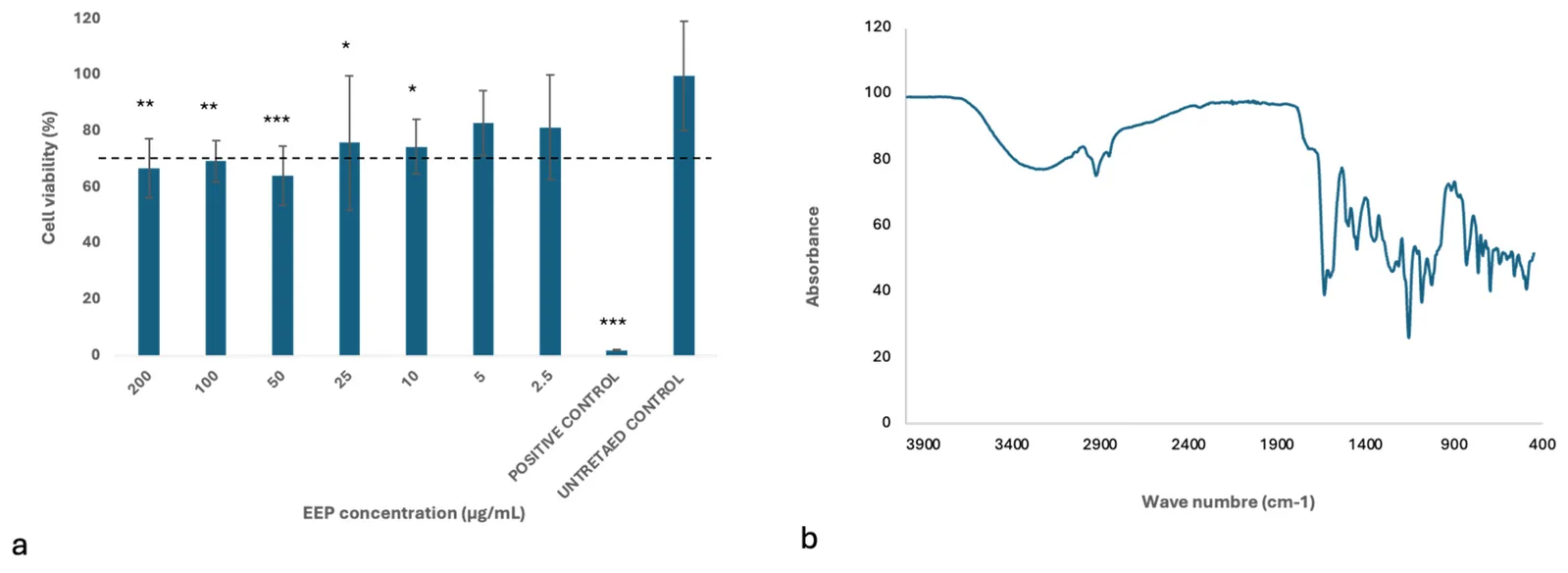
(**a**) Cell viability of NIH-3T3 fibroblasts after 24 h exposure to EEP (2.5–200 µg/mL), assessed by resazurin-based assay. Data expressed as mean ± SD (n = 6) normalized to the untreated control (100%). * *p* < 0.05; ** *p* < 0.01; *** *p* < 0.001. Dashed line: 70% viability threshold (ISO 10993-5). (**b**) FTIR-ATR spectrum of EEP (4000–400 cm^−1^).

The effect of EEP on NIH-3T3 fibroblasts was evaluated using a resazurin-based metabolic assay after 24 h exposure to concentrations ranging from 2.5 to 200 µg/mL (Figure 2a). Cell viability remained above 60% across all tested concentrations. According to the ISO 10993-5 [25]. criterion used in this study, concentrations yielding cell viability ≥ 70% are considered non-cytotoxic; under this criterion, concentrations ≤ 25 µg/mL met the threshold, whereas higher concentrations reduced viability to values below 70%.

#### 3.1.2. Fourier Transform Infrared Spectroscopy (FTIR-ATR)

The FTIR-ATR spectrum of the EEP is shown in Figure 2b. A broad band around 3200–3500 cm^−1^ corresponds to O–H stretching vibrations of hydroxyl groups present in phenolic structures. Bands at 2924 and 2854 cm^−1^ are attributed to asymmetric and symmetric C–H stretching of aliphatic groups, respectively. Signals at 1635 cm^−1^ are associated with C=O stretching and aromatic C=C vibrations, characteristic of flavonoids and polyphenolic compounds. Absorption bands at 1157 and 1083 cm^−1^ correspond to C–O stretching vibrations of alcohols and ethers.

### 3.2. Physicochemical and Biological Evaluation of Propolis-Based Hydrogels

#### 3.2.1. Macroscopic Characterization

**Figure 3:**
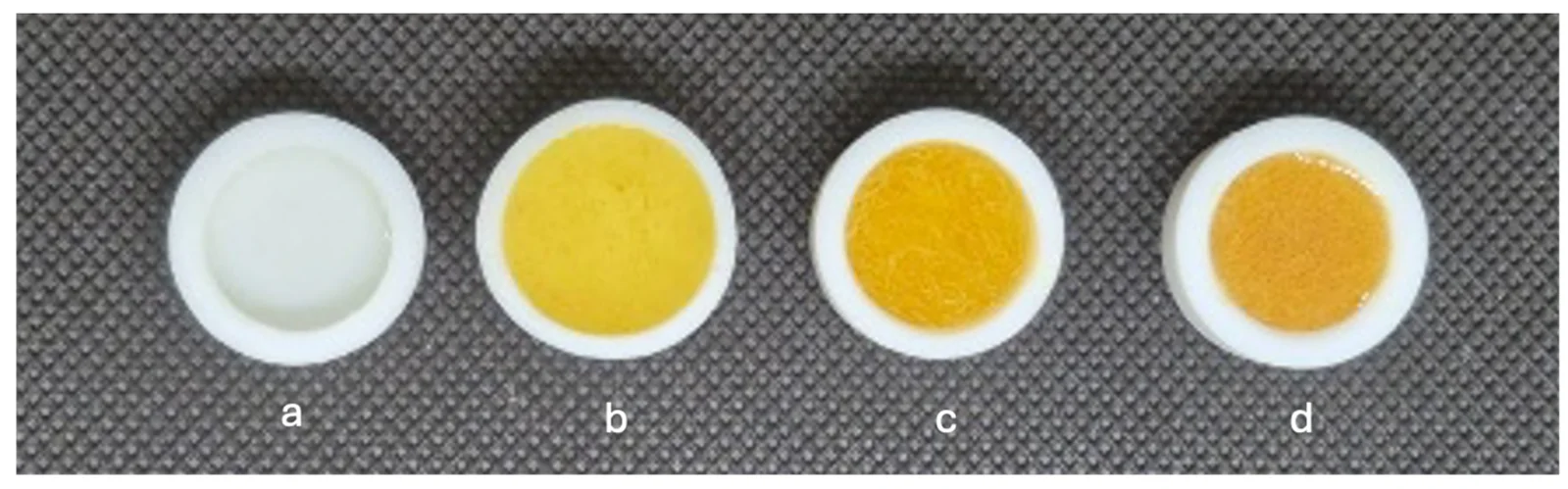
Macroscopic appearance of chitosan–propolis hydrogels in their hydrated state: (**a**) propolis-free chitosan hydrogel (control), (**b**) hydrogel containing 1% (*w*/*w*) EEP, (**c**) hydrogel containing 3% (*w*/*w*) EEP, and (**d**) hydrogel containing 5% (*w*/*w*) EEP.

The macroscopic appearance of the hydrated chitosan–propolis hydrogels is shown in Figure 3. The propolis-free chitosan hydrogel (Figure 3a) exhibited a whitish translucent appearance. Hydrogels containing EEP displayed a progressive change in coloration with increasing extract concentration. The hydrogel containing 1% (*w*/*w*) EEP (Figure 3b) presented a pale-yellow color, while those containing 3% and 5% (*w*/*w*) EEP (Figure 3c,d) exhibited progressively more intense yellow-orange colorations.

#### 3.2.2. Release, Degradation, Swelling, and Antioxidant Activity

**Figure 4:**
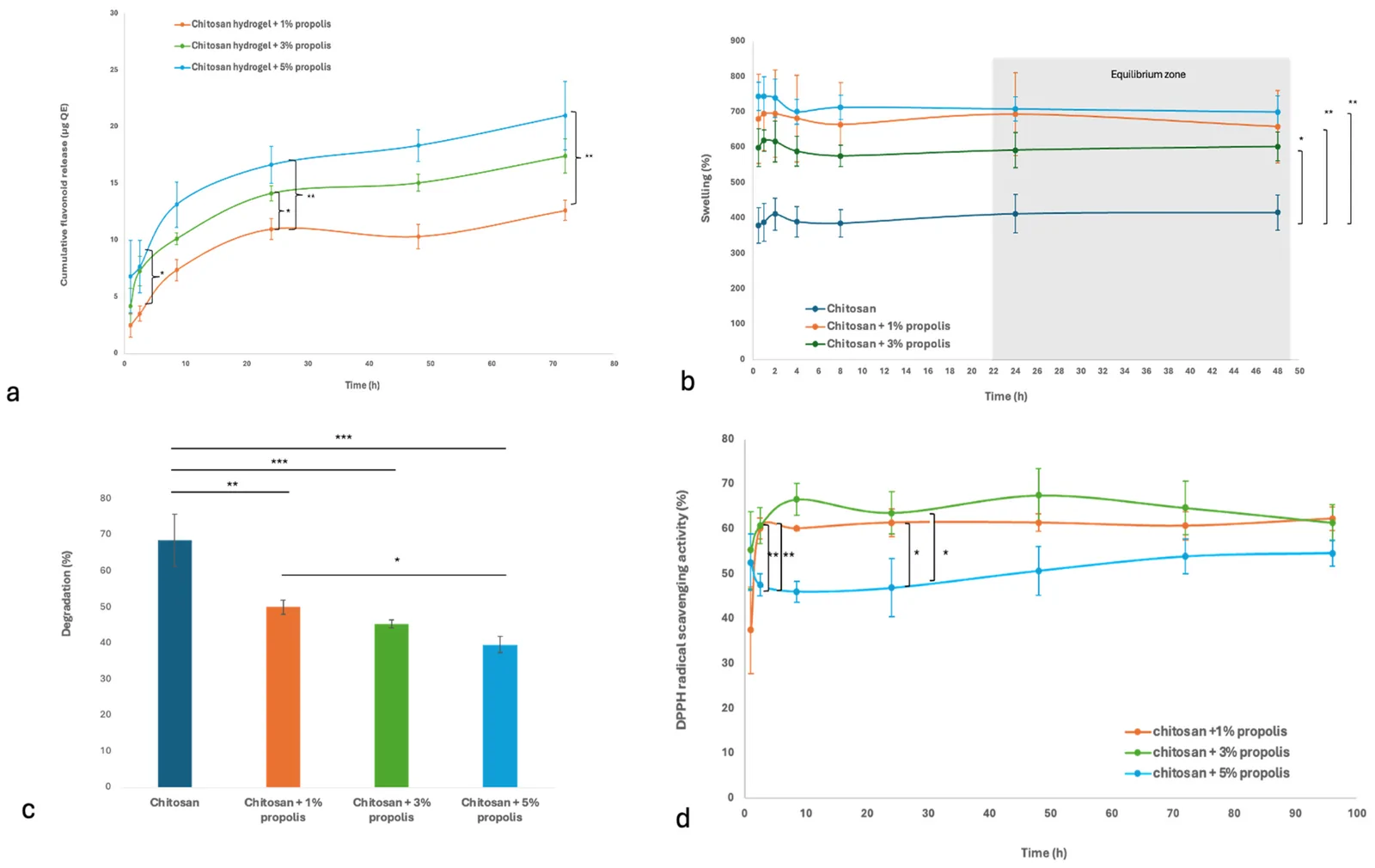
(**a**) Cumulative flavonoid release (µg quercetin equivalents, QE) from lyophilized chitosan–propolis hydrogels containing 1%, 3%, and 5% (*w*/*w*) EEP over 72 h. (**b**) Swelling profiles of chitosan–propolis hydrogels over 48 h. (**c**) Mass loss (%) after 16 days. (**d**) DPPH radical scavenging activity (%) of compounds released from chitosan–propolis hydrogels over 96 h. The propolis-free chitosan hydrogel showed no detectable antioxidant activity and was therefore excluded from panel d. For release, swelling, and degradation assays, samples were maintained in PBS (pH 7.4) at 37 °C; DPPH reactions were performed at room temperature. Data are presented as mean ± SD (n = 3). Brackets indicate statistically significant pairwise comparisons (* *p* < 0.05, ** *p* < 0.01, *** *p* < 0.001). EEP: ethanolic extract of propolis.

The flavonoid release profile (Figure 4a) showed an initial release phase followed by a more gradual and sustained release over 72 h. Cumulative flavonoid release increased with EEP content, reaching 12.64 ± 0.90, 17.44 ± 1.50, and 21.01 ± 3.03 µg QE for the 1%, 3%, and 5% (*w*/*w*) formulations, respectively. These values represent total flavonoid-equivalent release under the experimental conditions and should not be interpreted as tissue concentrations or therapeutic doses. Significant differences were observed between the 1% and 5% EEP formulations at all evaluated time points (*p* ≤ 0.042). All hydrogels demonstrated high water absorption capacity (Figure 4b), with rapid swelling occurring within the first 2 h, followed by relatively stable swelling values up to 48 h. The propolis-free chitosan hydrogel showed the lowest swelling value (414.88 ± 51.60%), whereas all EEP-containing formulations exhibited significantly higher swelling (677.03 ± 110.00%, 598.23 ± 45.16%, and 705.70 ± 39.83% for the 1%, 3%, and 5% EEP formulations, respectively; *p* < 0.05 vs. chitosan), with no significant differences among EEP concentrations.

Degradation after 16 days (Figure 4c) was highest in the chitosan control (68.54 ± 7.30%) and progressively decreased with increasing EEP content (50.12 ± 1.96%, 45.38 ± 1.14%, and 39.65 ± 2.30% for the 1%, 3%, and 5% EEP formulations, respectively; *p* < 0.001 vs. chitosan). Regarding antioxidant activity (Figure 4d), the chitosan control showed no detectable DPPH radical-scavenging activity, whereas all EEP-containing formulations exhibited sustained antioxidant activity throughout the 96 h evaluation period. The 1% and 3% formulations showed comparable activity at 96 h (~62%), whereas the 5% EEP formulation exhibited significantly lower activity at early time points (*p* < 0.05 vs. 1% and 3% EEP at 2.5 and 24 h), with differences diminishing over time and activity reaching 54.7 ± 2.9% at 96 h.

#### 3.2.3. Morphological Characterization

**Figure 5:**
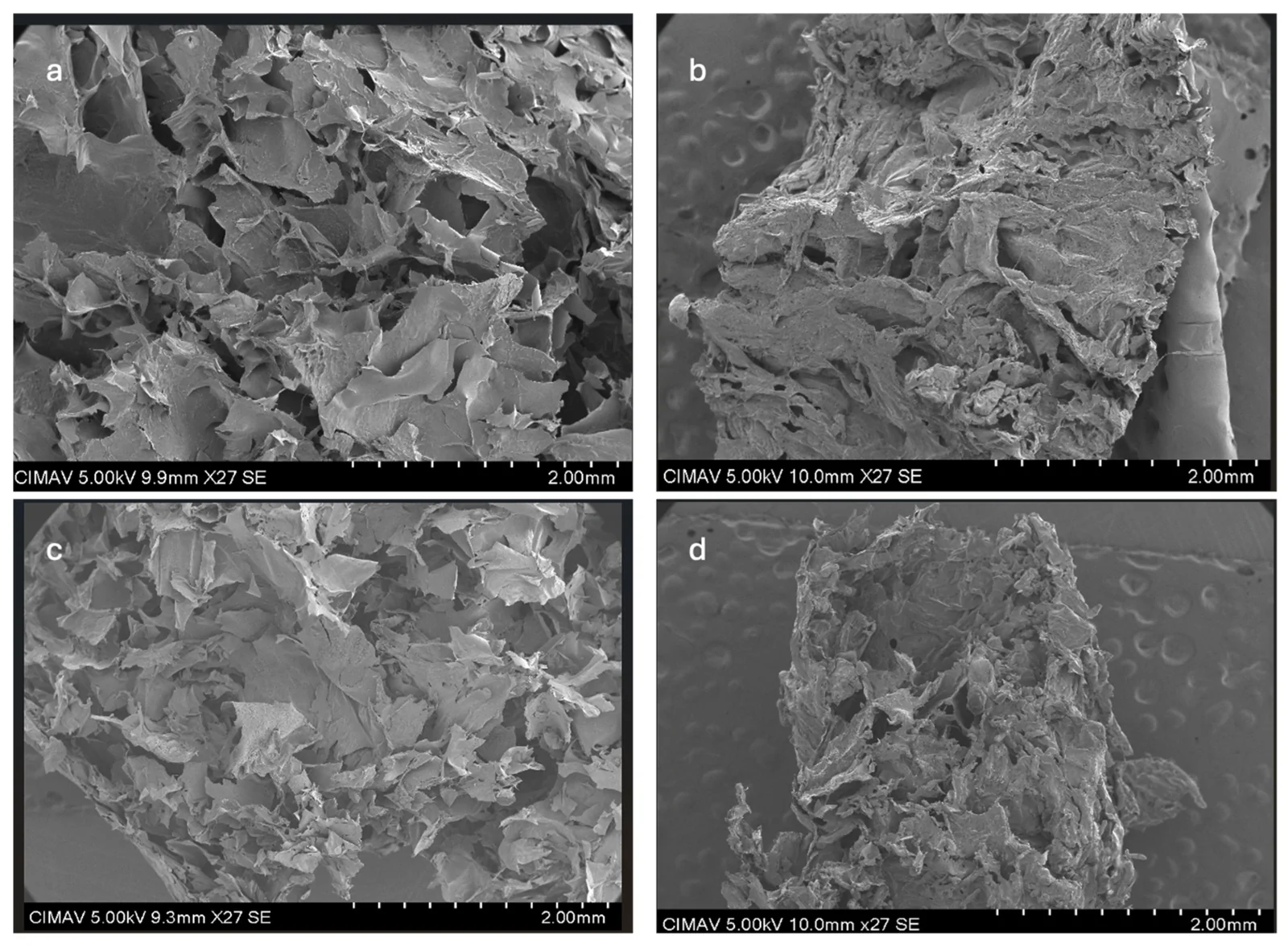
Scanning electron microscopy (SEM) micrographs of lyophilized chitosan–propolis hydrogels obtained by cryogenic fracture: (**a**) propolis-free chitosan hydrogel (control), (**b**) hydrogel containing 1% (*w*/*w*) EEP, (**c**) hydrogel containing 3% (*w*/*w*) EEP, and (**d**) hydrogel containing 5% (*w*/*w*) EEP. Images were acquired at 5.0 kV accelerating voltage, 27× magnification. Scale bar = 2.00 mm.

The surface and cross-sectional morphology of the lyophilized chitosan–propolis hydrogels was examined by SEM as shown in Figure 5. The propolis-free chitosan hydrogel (Figure 5a) exhibited a characteristic lamellar architecture consisting of flat, overlapping sheets with well-defined edges and broad interlamelar spaces, resulting in an open and highly porous three-dimensional network. Upon incorporation of EEP, concentration-dependent modifications in the microstructural organization were observed. The hydrogel containing 1% (*w*/*w*) EEP (Figure 5b) displayed a noticeably more compact arrangement, with thicker and partially rolled lamellae, a marked reduction in interlamellar spaces, and a rougher surface texture compared to the control. Additional material was observed adhered between the lamellae, suggesting partial filling of the porous network by propolis constituents. At 3% (*w*/*w*) EEP (Figure 5c), the lamellar organization was partially preserved but with increased fragmentation and heterogeneity in the distribution of interlamellar spaces, indicating a structural transition toward greater disorder. The hydrogel containing 5% (*w*/*w*) EEP (Figure 5d) presented the most markedly altered morphology, characterized by densely stacked and thickened lamellae, a substantial reduction in interlamellar porosity, and a predominantly columnar arrangement in certain regions, reflecting a denser and more heterogeneous microarchitecture overall.

#### 3.2.4. Thermogravimetric and Differential Scanning Calorimetry Analysis

**Figure 6:**
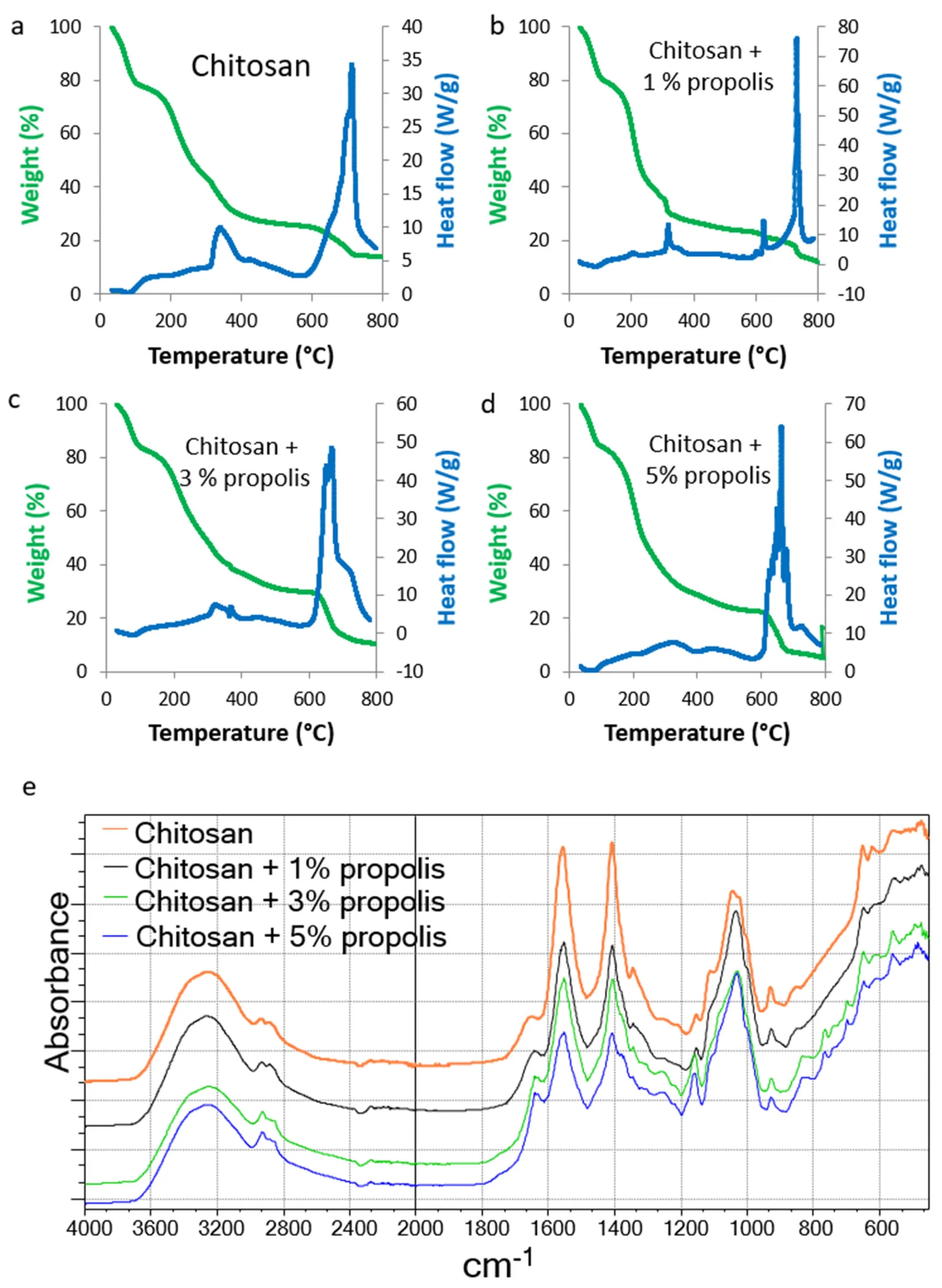
Simultaneous thermogravimetric analysis (TGA) and differential scanning calorimetry (DSC) curves of chitosan–propolis hydrogels: (**a**) propolis-free chitosan hydrogel, (**b**) hydrogel containing 1% (*w*/*w*) EEP, (**c**) hydrogel containing 3% (*w*/*w*) EEP, and (**d**) hydrogel containing 5% (*w*/*w*) EEP. Green curves represent weight loss (%) and blue curves represent heat flow (W/g) as a function of temperature (30–800 °C, heating rate 10 °C/min, air atmosphere). (**e**) FTIR-ATR spectra of the propolis-free chitosan hydrogel and chitosan–propolis hydrogels containing 1%, 3%, and 5% (*w*/*w*) EEP, recorded over the wavenumber range of 4000–450 cm^−1^.

The thermal behavior of the chitosan–propolis hydrogels is shown in Figure 6a–d. All TGA curves exhibited a progressive decrease in mass as temperature increased, with three well-defined decomposition stages. The first stage, occurring between 25 and 150 °C, corresponded to the loss of adsorbed and bound water, with mass losses ranging from 19.29% (3% EEP) to 23.91% (chitosan), and DTG peak temperatures between 67 and 75 °C across all formulations. The second stage, between 150 and 400 °C, represented the most significant mass loss event, attributed to the thermal decomposition of the chitosan backbone and propolis constituents, with losses of 46.46%, 49.81%, 43.71%, and 50.10% for the chitosan, 1%, 3%, and 5% (*w*/*w*) EEP formulations, respectively. The third stage, between 400 and 800 °C, corresponded to the combustion of residual organic matter and was notably more pronounced in the propolis-loaded formulations, particularly in the 3% EEP (26.28%) and 5% EEP (23.49%) groups compared to the chitosan control (15.46%). The residual mass at 800 °C decreased progressively with increasing EEP content: 14.15% for the propolis-free chitosan hydrogel, 12.49%, 10.69%, and 5.39% for the 1%, 3%, and 5% (*w*/*w*) EEP formulations, respectively.

The corresponding DSC curves revealed distinct thermal events among the formulations. The propolis-free chitosan hydrogel (Figure 6a) displayed a broad exothermic event at 338.7 °C and a high-temperature peak at 711.9 °C. In contrast, the propolis-loaded formulations (Figure 6b–d) exhibited additional exothermic peaks and increased signal intensity, particularly in the high-temperature region. The 1% EEP hydrogel showed peaks at 203.5, 317.1, and 600.0 °C, while the 3% EEP formulation presented prominent events at 320.4, 367.4, and 649.3–665.3 °C, the latter with the highest heat flow intensity observed across all groups (up to 617.6 mW). The 5% EEP hydrogel showed exothermic peaks at 320.1, 447.4, and 624.6–660.7 °C. The number and intensity of DSC events increased with propolis content, reflecting the progressive incorporation of thermally active propolis constituents into the chitosan matrix.

#### 3.2.5. FTIR-ATR Spectroscopic Analysis

The FTIR-ATR spectra of the lyophilized chitosan–propolis hydrogels are shown in Figure 6e. All formulations displayed characteristic absorption bands common to chitosan, including a broad band in the 3250–3266 cm^−1^ region corresponding to O–H and N–H stretching vibrations, bands at 2926–2932 cm^−1^ attributed to C–H stretching, and multiple peaks in the fingerprint region (1800–450 cm^−1^). Differences among the spectra were observed primarily in band intensity and in the appearance of new bands with increasing propolis content. Notably, a shift in the C–O–C stretching band from 1040 cm^−1^ in the propolis-free chitosan hydrogel to 1028–1034 cm^−1^ in the EEP-containing formulations was identified, along with new bands at 1153–1159 cm^−1^ and at 555 and 646 cm^−1^ in the 3% and 5% (*w*/*w*) EEP hydrogels, which were absent in the chitosan control.

#### 3.2.6. Antimicrobial Activity

**Figure 7:**
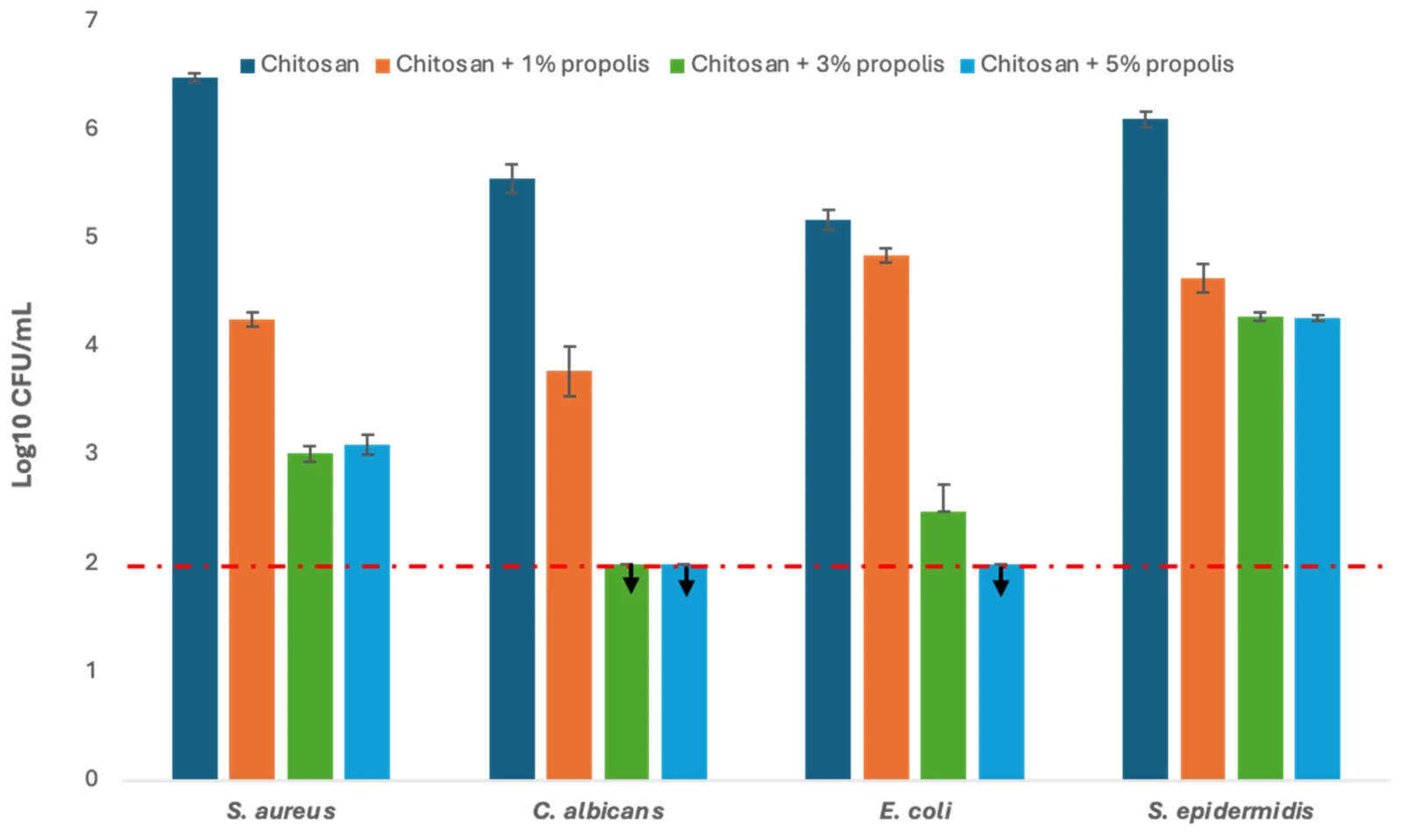
Microbial load (log_10_ CFU/mL) recovered from chitosan–propolis hydrogels after 24 h of contact with each microbial suspension, evaluated by the drop plate method. Bars represent mean log_10_ CFU/mL (n = 3). Red dashed line indicates the detection limit (10^2^ CFU/mL; log_10_ = 2.0). Downward arrows indicate values below the detection limit (<10^2^ CFU/mL).

The antimicrobial activity of the chitosan–propolis hydrogels was evaluated by two complementary methods: the agar disc diffusion assay and the drop plate method. Results are presented in Table 2 and Figure 7, respectively. The propolis-free chitosan hydrogel showed no inhibition zones against any of the tested microorganisms in the agar disc diffusion assay, and microbial loads comparable to the inoculum were recovered by the drop plate method across all strains. In contrast, all EEP-containing formulations exhibited concentration-dependent antimicrobial activity against *Staphylococcus aureus*, *Staphylococcus epidermidis,* and *Candida albicans*. For *S. aureus*, inhibition zones increased progressively with EEP concentration, ranging from 10.7 ± 1.5 mm (1% *w*/*w* EEP) to 14.3 ± 3.2 mm (3% *w*/*w* EEP) and 17.0 ± 2.0 mm (5% *w*/*w* EEP). The drop plate method confirmed a reduction in viable counts with increasing propolis content, with log_10_ CFU/mL values of 6.49, 4.26, 3.03, and 3.10 for the chitosan control, 1%, 3%, and 5% (*w*/*w*) EEP formulations, respectively. For *S. epidermidis*, inhibition zones of 9.3 ± 1.5, 12.3 ± 2.5, and 14.3 ± 2.9 mm were recorded for the 1%, 3%, and 5% (*w*/*w*) EEP hydrogels, respectively. The drop plate method showed a reduction in microbial load from 6.11 log_10_ CFU/mL (chitosan control) to 4.64, 4.28, and 4.27 log_10_ CFU/mL for the 1%, 3%, and 5% (*w*/*w*) EEP formulations, respectively. The highest inhibitory activity was observed against *C. albicans*, with inhibition zones of 11.0 ± 2.8, 16.0 ± 1.4, and 20.0 ± 4.2 mm for the 1%, 3%, and 5% (*w*/*w*) EEP hydrogels, respectively. The drop plate method showed a reduction from 4.67 log_10_ CFU/mL in the chitosan control to 3.78 log_10_ CFU/mL for the 1% EEP formulation, while no viable colonies were detected for the 3% and 5% (*w*/*w*) EEP hydrogels (<10^2^ CFU/mL). No inhibition zones were detected against *Escherichia coli* O157:H7 in the agar disc diffusion assay for any formulation. However, the drop plate method revealed a reduction in viable counts with increasing EEP concentration, with log_10_ CFU/mL values of 5.22, 4.85, and 2.48 for the chitosan control, 1%, and 3% (*w*/*w*) EEP formulations, respectively, and no viable colonies detected for the 5% (*w*/*w*) EEP hydrogel (<10^2^ CFU/mL).

#### 3.2.7. Cell Viability Evaluation of Hydrogel-Conditioned Media

**Figure 8:**
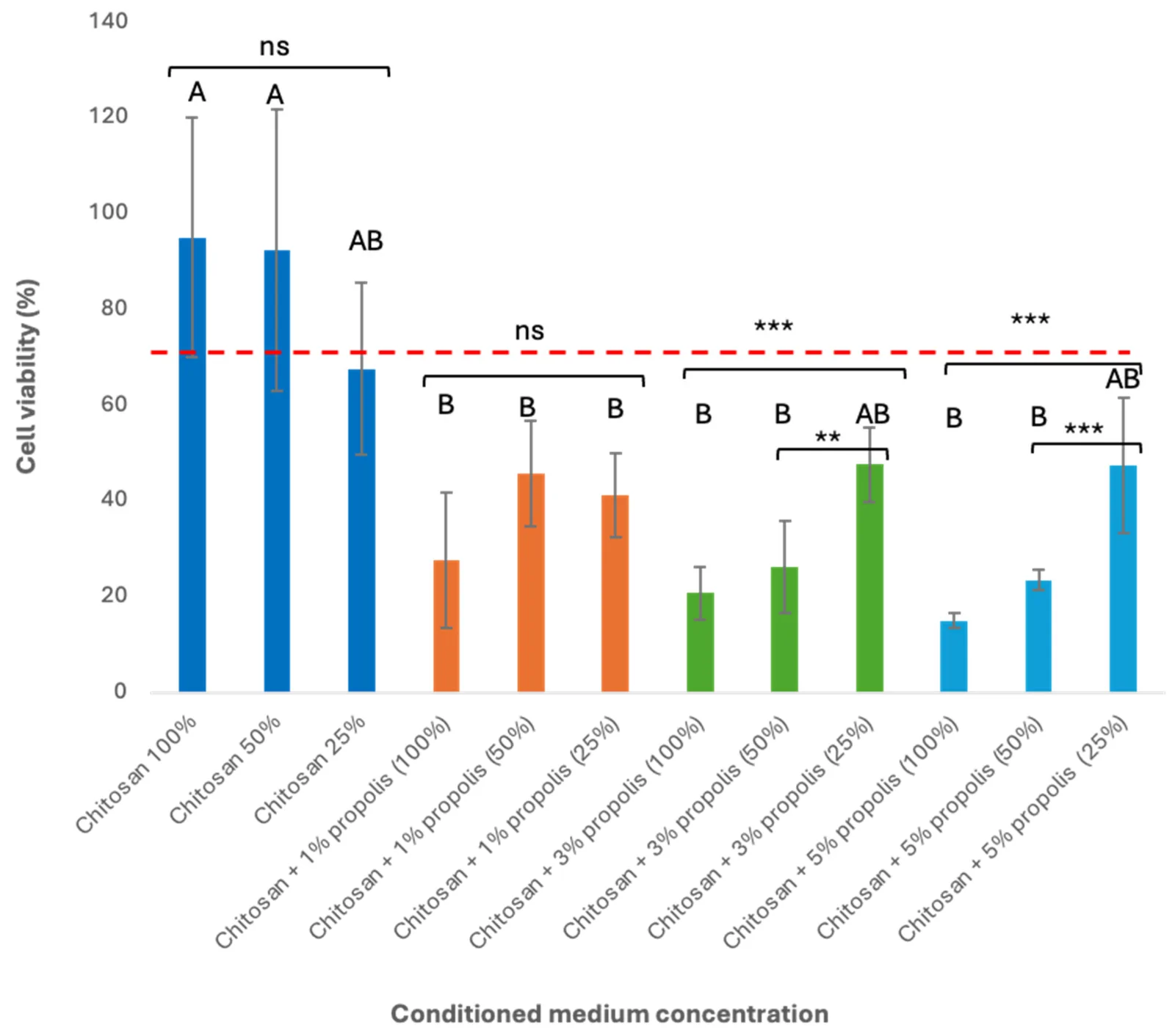
Cell viability of NIH-3T3 murine fibroblasts exposed to hydrogel-conditioned media using a resazurin-based metabolic assay. Cell viability is expressed as percentage relative to the untreated control. Bars represent mean ± SD (n = 6). The dashed line indicates the 70% viability criterion used for interpretation according to ISO 10993-5. Uppercase letters above bars indicate statistically significant differences among groups at the same conditioned medium concentration; groups sharing a letter are not significantly different (*p* > 0.05). Brackets indicate statistically significant differences between conditioned medium concentrations within the same formulation: ** *p* < 0.01; *** *p* < 0.001.

Cell viability after exposure to hydrogel-conditioned media was evaluated in NIH-3T3 fibroblasts, and the results are shown in Figure 8. The positive cytotoxicity control yielded mean viability values of 9.12 ± 0.18% and 9.82 ± 0.81%, confirming assay validity. Cells exposed to conditioned medium from the propolis-free chitosan hydrogel exhibited high viability at 100% and 50% concentrations (91.91 ± 36.15% and 93.74 ± 48.26%, respectively), both above the 70% non-cytotoxicity threshold established by ISO 10993-5, with a slight decrease at 25% dilution (63.65 ± 20.51%). In contrast, conditioned media from EEP-containing formulations showed markedly reduced cell viability across all tested concentrations, with values consistently below the ISO 10993-5 threshold. A concentration-dependent trend was observed for all propolis-loaded formulations, with cell viability increasing as the conditioned medium was diluted. The 1% (*w*/*w*) EEP hydrogel showed viabilities of 25.10 ± 8.33%, 47.35 ± 25.71%, and 39.42 ± 12.78% at 100%, 50%, and 25% conditioned medium, respectively. The 3% (*w*/*w*) EEP formulation yielded values of 21.16 ± 10.60%, 25.38 ± 11.43%, and 47.09 ± 4.29%, and the 5% (*w*/*w*) EEP hydrogel exhibited the lowest viability at 100% conditioned medium (14.94 ± 1.22%), with a gradual increase at lower concentrations (23.45 ± 2.19% at 50% and 47.36 ± 13.27% at 25%). Statistical analysis revealed significant differences among groups at all tested dilutions (100%: F = 20.87, *p* < 0.001; 50%: F = 8.20, *p* < 0.001; 25%: F = 3.21, *p* = 0.045), with the chitosan control showing significantly higher viability than all EEP-containing formulations at 100% and 50% (*p* < 0.05). No significant differences were observed among the three EEP formulations at any concentration (*p* > 0.05).

#### 3.2.8. Wound Contraction

**Figure 9:**
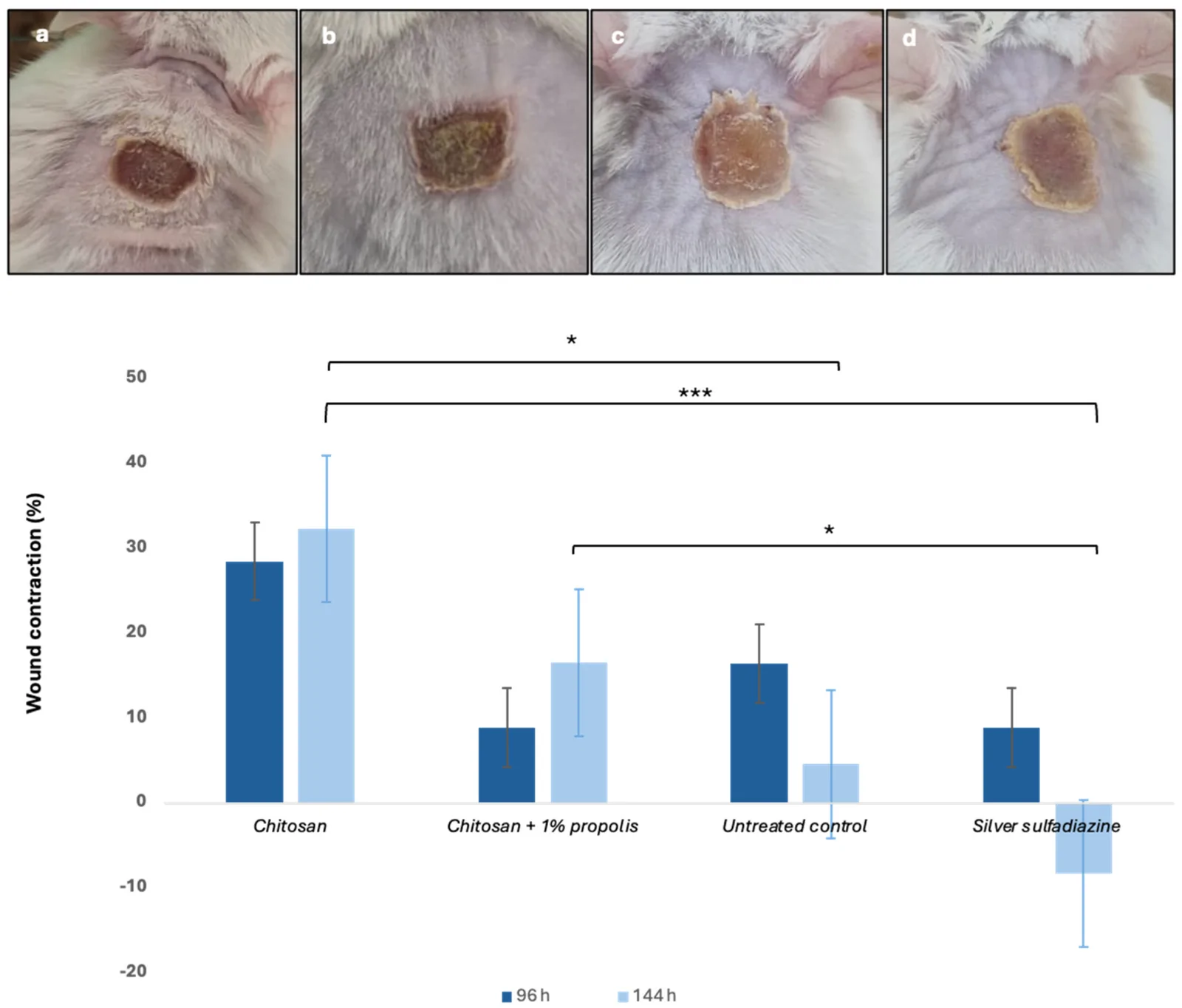
Clinical evaluation of burn wound healing in the murine second-degree burn model. Upper panel: representative photographs of burn wounds at 144 h post-burn induction: (**a**) chitosan hydrogel, (**b**) 1% (*w*/*w*) EEP chitosan hydrogel, (**c**) untreated control, and (**d**) silver sulfadiazine. Lower panel: percentage of wound contraction at 96 and 144 h post-burn induction. Bars represent mean ± SD (n = 6 per group). Brackets indicate statistically significant differences (* *p* < 0.05; *** *p* < 0.001).

Wound contraction profiles for all experimental groups at 96 and 144 h post-burn induction are shown in Figure 9. At 96 h, no statistically significant differences in wound contraction were observed among the four experimental groups. At 144 h, statistically significant differences among groups were detected (*p* < 0.001). The chitosan hydrogel group exhibited the highest wound contraction, significantly greater than both the silver sulfadiazine group (*p* < 0.001) and the untreated control group (*p* = 0.018). The 1% (*w*/*w*) EEP hydrogel group showed intermediate contraction, significantly greater than the silver sulfadiazine group (*p* = 0.021) but not significantly different from the chitosan hydrogel or the untreated control (*p* > 0.05). Notably, the silver sulfadiazine group was the only group to present net wound expansion at 144 h, with no significant difference compared to the untreated control (*p* > 0.05).

#### 3.2.9. Histological Evaluation

**Figure 10:**
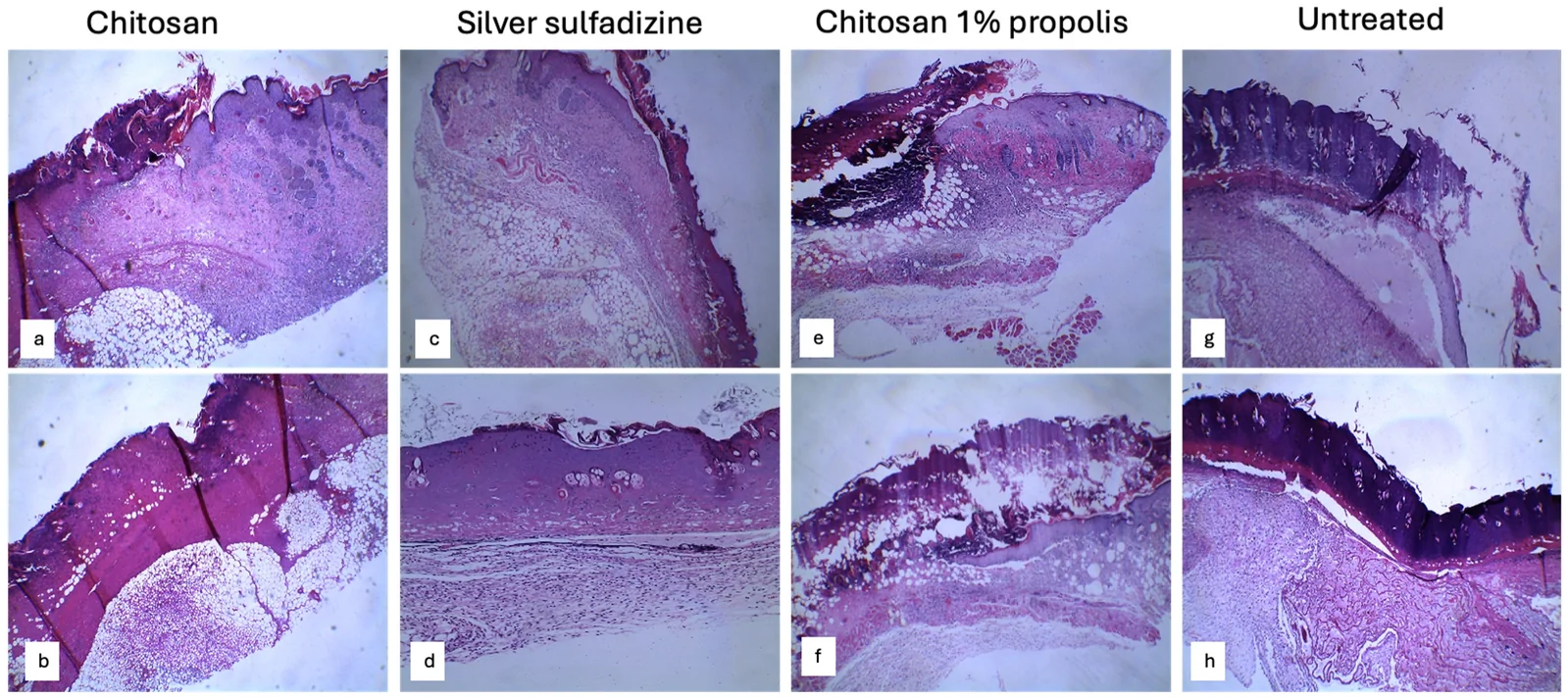
Representative histological sections of burn wounds stained with hematoxylin and eosin (H&E) and examined at 10× magnification. (**a**) chitosan hydrogel group, wound-edge region; (**b**) chitosan hydrogel group, central lesion region; (**c**) silver sulfadiazine group, wound-edge region; (**d**) silver sulfadiazine group, central lesion region; (**e**) 1% EEP-loaded chitosan hydrogel group, wound-edge region; (**f**) 1% EEP-loaded chitosan hydrogel group, central lesion region; (**g**) untreated control group, wound-edge region; (**h**) untreated control group, central lesion region. Histological observations were descriptive and representative of the overall tissue response observed within each experimental group.

Representative H&E-stained sections examined at 10× magnification showed distinct tissue features among the experimental groups (Figure 10). In the chitosan hydrogel group, the wound-edge region (a) exhibited a keratinized stratified squamous epithelium composed of approximately 12–20 cell layers. The underlying dermis contained abundant mature collagen fibers arranged in an irregular and disorganized pattern, accompanied by mild chronic inflammatory infiltrate and discrete angiogenesis, findings consistent with early tissue remodeling. In the central lesion area (b), epithelial discontinuity consistent with ulceration was observed, covered by a dense fibrin layer, while the underlying connective tissue showed diffuse mixed inflammatory infiltrate and abundant adipose tissue in the basal region. In the silver sulfadiazine group, the wound-edge region (c) showed a keratinized stratified squamous epithelium with focal areas of hyperkeratosis. The dermis contained abundant mature collagen fibers with an irregular arrangement and diffuse mixed inflammatory infiltrate. Hair follicles and sebaceous glands were identified within the connective tissue. In the central lesion area (d), epithelial loss consistent with ulceration was observed, with fibrin deposition on the surface. A thick layer of coagulative necrosis remained attached to the lesion, whereas in other regions the necrotic tissue had detached. The underlying connective tissue exhibited myxoid changes, diffuse mixed inflammatory infiltrate, and a limited amount of adipose tissue in the basal region. In the 1% EEP-loaded chitosan hydrogel group, the wound-edge region (e) displayed epithelial discontinuity covered by a thick layer of coagulative necrosis associated with fibrin deposits and hemorrhagic debris. In contrast, the distal region, corresponding to the active wound margin, demonstrated early re-epithelialization characterized by newly formed epithelial extensions, incipient granulation tissue, and moderate diffuse mixed inflammatory infiltrate. Hair follicles and glandular structures were identified in several sections examined from this treatment group. Although these structures may not be clearly visible in the representative micrograph shown, they were observed in other sections from animals within the group. Abundant adipose tissue was also present in the basal region. In the central lesion area (f), extensive ulceration was observed, covered by fibrin and a thick layer of coagulative necrotic tissue. The underlying dermis contained adipose tissue, muscle fibers, and loose myxoid connective tissue associated with diffuse mixed inflammatory infiltrate and evidence of early angiogenesis. In the untreated control group, the wound-edge region (g) exhibited stratified squamous epithelium with atrophic epithelial projections and the presence of a subepithelial blister. Hair follicles and glandular structures were less frequently observed in the examined sections. The connective tissue displayed disorganized collagen fibers and moderate-to-severe diffuse mixed inflammatory infiltrate. Loose connective tissue containing minimal adipose tissue and smooth muscle fibers was observed in deeper regions, with marked disruption of normal tissue architecture. In the central lesion area (h), extensive epithelial discontinuity was observed, with surface fibrin deposition and coagulative necrosis. The underlying connective tissue was extensively necrotic, with no evidence of viable tissue or adipose tissue at the base of the lesion, findings consistent with a severe necrotic injury.

Overall, the histological findings were compatible with different stages of early tissue repair among the experimental groups. The untreated control group showed extensive necrosis and marked disruption of tissue architecture, whereas the chitosan hydrogel and silver sulfadiazine groups showed features compatible with early repair, including partial re-epithelialization, collagen deposition, and tissue remodeling. The 1% EEP-loaded chitosan hydrogel group also showed features compatible with early repair, and cutaneous appendages were observed in several examined sections. Because the histological assessment was descriptive and not quantitatively scored, these observations should be considered preliminary and should not be interpreted as evidence of enhanced regeneration or preservation of cutaneous appendages.

## 4. Discussion

The present study reports the development and exploratory in vivo evaluation of a chitosan-based hydrogel enriched with EEP originating from Chihuahua, Mexico, characterized using parameters established in NOM-003-SAG/GAN-2017, and assessed in a murine model of second-degree burns.

The qualitative reactions specified by NOM-003-SAG/GAN-2017 were positive for phenolic and flavonoid compounds. These preliminary findings were followed by quantitative determinations, which yielded a total phenolic content of 27.42 ± 2.53% GAE and a total flavonoid content of 9.23 ± 0.314% QE. The EEP from apiaries in Cuauhtémoc, Chihuahua, met the NOM-003-SAG/GAN-2017 thresholds for total phenolic content (27.42 ± 2.53% gallic acid equivalents; minimum: ≥5%) and total flavonoid content (9.23 ± 0.314% quercetin equivalents; minimum: ≥0.5%). These values exceeded the regulatory minimums and were comparable to those reported for Indian propolis by Kapare et al. [26] and Egyptian propolis by Saleh et al. [27]. The present study did not include chromatographic identification of individual constituents; therefore, the observed biological activities cannot be attributed to specific phenolic or flavonoid compounds in the Chihuahua propolis sample. The geographic provenance of propolis remains relevant because composition may vary among regions and sources [17,28], and seasonal and climatic conditions may further contribute to quantitative variability in its phytochemical composition. However, the present data do not establish a unique regional phytochemical profile. Previous studies have reported biological activity of Chihuahua propolis [29,30], while other chitosan–propolis biomaterials have been evaluated using propolis from different sources [31,32,33]. Thus, the contribution of the present study lies in the integrated characterization of a geographically defined Chihuahua propolis collected in September 2024, incorporated into a chitosan hydrogel and evaluated across physicochemical, functional, and exploratory in vivo endpoints.

An apparent discrepancy was observed between the high total phenolic and flavonoid contents of the EEP and its moderate DPPH radical-scavenging activity (IC_50_ = 184.00 ± 9.70 µg/mL), which did not meet the NOM-003-SAG/GAN-2017 threshold (≤100 µg/mL), while the oxidation index (31.33 ± 6.11 s) exceeded the regulatory maximum of 22 s. These findings are not necessarily contradictory because DPPH activity depends on the qualitative composition and structural characteristics of the compounds present, rather than on total phenolic or flavonoid content alone [34,35]. However, because the present study did not identify individual constituents by HPLC-MS or another compound-specific method, the observed antioxidant profile cannot be attributed to particular flavonoids or phenolic compounds. The current Mexican standard provides defined quality parameters for propolis, but it does not provide individual compound identification [19]. The DPPH assay also captures only specific radical-scavenging processes and does not encompass all possible antioxidant mechanisms [36]. Accordingly, the moderate DPPH activity should be interpreted as a property of the extract under the assay conditions rather than as evidence of a specific phytochemical mechanism. Comprehensive chromatographic characterization is warranted in future studies.

These findings reinforce the complexity of propolis as a multicomponent natural product and the fact that total phenolic and flavonoid measurements do not by themselves predict all biological activities. The moderate free-extract DPPH activity did not preclude sustained radical-scavenging activity in the hydrogel release medium, which exhibited 54–62% DPPH inhibition over 96 h. The maximum cumulative flavonoid release of 21.01 ± 3.03 µg QE at 72 h represents a total flavonoid-equivalent value under the experimental conditions rather than a concentration of an individual compound at the wound site; therefore, its therapeutic adequacy cannot be judged from the present release data alone. This finding is consistent with the release of antioxidant-active compounds from the EEP-containing hydrogels under the experimental conditions [31]. However, the present study did not determine the identity of the released compounds, their tissue deposition, permeation, or local bioavailability, and therefore the release data should be interpreted as comparative evidence of sustained release and antioxidant activity rather than as proof of a specific mechanism.

The *Artemia salina* lethality assay yielded an LC_50_ of 26.40 ± 4.75 µg/mL, indicative of high toxicity according to the Meyer et al. classification [37]. This result should nevertheless be interpreted cautiously because *A. salina* is an invertebrate screening model and does not directly predict mammalian toxicity in a topical application. The assay exposes nauplii directly to the test extract without the delivery matrix used in the hydrogel. The negligible hemolytic activity (0.006 ± 0.0039%) provides additional information regarding erythrocyte lysis under the tested conditions, but it does not establish general biocompatibility. Likewise, NIH-3T3 viability remained above 60% at all tested EEP concentrations and reached the 70% ISO 10993-5 criterion at concentrations ≤ 25 µg/mL. Taken together, these results indicate that the EEP presents different responses across screening models and support the need for further, physiologically relevant safety evaluation rather than allowing a conclusion of mammalian safety.

The cellular response to free EEP and the hydrogel formulations should be interpreted in the context of their different exposure conditions. The conditioned-medium approach, based on the extract method principle of ISO 10993-5, generates a static and relatively concentrated eluate that does not reproduce the diffusion, fluid turnover, or tissue clearance occurring in a wound environment [38]. Under the tested conditions, the propolis-free chitosan hydrogel showed viability above 70% at 100% and 50% conditioned medium, whereas all EEP-containing formulations showed values below this criterion. However, the resazurin assay primarily reflects cellular metabolic activity and cannot distinguish apoptosis, necrosis, or reversible cytostatic/metabolic effects [39]. Therefore, these findings should be interpreted as reduced metabolic activity/viability under the specific extraction and exposure conditions rather than as evidence of a defined cellular mechanism or definitive cytotoxicity [38,39]. Similar initial reductions in in vitro viability have been reported for propolis-loaded scaffolds under static conditions, including chitosan/collagen and PVA-based systems [40], while Moreno et al. [41] reported metabolic recovery and increased cell proliferation after longer exposure. The absence of significant differences among the EEP-containing formulations also does not support the conclusion that the 1% formulation is safer than the 3% or 5% formulations. Importantly, the short-term in vivo findings provide complementary evidence but should not be interpreted as confirmation of biocompatibility or absence of cytotoxicity. No overt treatment-related clinical signs were observed during the six-day period; however, longer follow-up and complementary assays are needed to determine cellular and tissue responses under more physiologically relevant conditions.

Physicochemical characterization showed that incorporation of EEP altered the spectral and thermal behavior of the chitosan matrix. FTIR-ATR analysis showed a shift in the C–O–C stretching band from 1040 to 1028–1034 cm^−1^ and the appearance of additional bands at 1153–1159, 555, and 646 cm^−1^. These spectral changes are consistent with interactions between chitosan and constituents of the extract, although the present analyses do not establish a specific molecular interaction or confirm molecular dispersion of individual propolis constituents [32]. The sustained flavonoid release observed over 72 h is consistent with retention and gradual release of extract-associated compounds from the hydrogel matrix. TGA and DSC analyses further showed changes in thermal behavior after EEP incorporation, including additional exothermic events and a progressive decrease in residual mass at 800 °C (from 14.15% in the chitosan control to 5.39% at 5% EEP), consistent with differences in the organic composition of the formulations.

SEM analysis revealed concentration-dependent microstructural modifications, from an open lamellar architecture in the chitosan control toward a progressively denser and more heterogeneous network with increasing EEP content. These changes were accompanied by increased swelling relative to the propolis-free control, with values reaching approximately 706%. The observed differences are consistent with modification of the matrix microstructure after EEP incorporation, although the present data do not establish a specific molecular mechanism for the changes in swelling. Comparable formulation-dependent physicochemical and biological behavior has been reported in other propolis-containing hydrogels [42]. Degradation decreased progressively with increasing EEP content (from 68.54% to 39.65% at 16 days), indicating greater mass retention under the conditions of the degradation assay. This behavior may be relevant to short-term topical applications, but it does not establish performance during the full wound-healing process.

Among the formulations evaluated in vitro, the 1% EEP hydrogel showed enhanced swelling relative to the propolis-free chitosan hydrogel, measurable flavonoid release, antioxidant activity in the release medium, antimicrobial activity, and a comparatively open morphology. These findings provided the experimental basis for selecting the 1% formulation for an initial exploratory in vivo evaluation. This selection should not be interpreted as evidence that 1% EEP is the optimal or therapeutically effective concentration, nor as evidence of superior cell viability relative to the higher EEP concentrations. The variability inherent to propolis sources further supports the need for characterization at each stage of formulation development.

The antimicrobial evaluation demonstrated a concentration-dependent response in all EEP-containing hydrogels against *S. aureus*, *S. epidermidis*, and *C. albicans*. No inhibition zones were detected against *E. coli* O157:H7 in the agar disc diffusion assay, consistent with the well-established selectivity of propolis phenolic constituents toward Gram-positive bacteria [43,44], as the outer membrane of Gram-negative organisms restricts passive diffusion of hydrophobic phenolics, a limitation also reported by Ferreira et al. [42] and Oliveira et al. [45] for propolis-containing hydrogels and supported by the fourfold higher MIC of the free EEP against *E. coli* (10.41 mg/mL) compared with *S. aureus* and *C. albicans* (2.60 mg/mL).

Critically, the drop plate method showed that the 5% EEP hydrogel reduced recoverable *E. coli* counts to below the detection limit (<10^2^ CFU/mL), despite the absence of an inhibition zone in the agar diffusion assay. This discrepancy between diffusion- and contact-based assays indicates that antimicrobial activity was more readily detected under direct-contact conditions for this organism. The findings are compatible with a contact-dependent contribution of the chitosan matrix and/or retained propolis constituents, but the present experiments do not establish a specific cooperative or synergistic mechanism between the two components. These results underscore the importance of complementary antimicrobial evaluation methods for contact-active biomaterials, as diffusion-based assays alone may underestimate antimicrobial effects [46,47].

From a clinical perspective, burn wounds are initially colonized predominantly by *S. aureus* and may subsequently acquire Gram-negative organisms and fungi, increasing the risk of wound infection and delayed healing [48]. A dressing capable of addressing this evolving microbial landscape through both diffusion-mediated and contact-dependent mechanisms could contribute meaningfully to infection control throughout the wound healing process.

The macroscopic wound contraction data at 144 h revealed significant superiority of both the propolis-free chitosan hydrogel and the 1% EEP hydrogel over silver sulfadiazine (*p* < 0.001 and *p* = 0.021, respectively), whereas the silver sulfadiazine group was the only group to show net wound expansion. These differences should be interpreted cautiously because the present study was not designed to establish the mechanism underlying the response to silver sulfadiazine, and the comparator was an ointment rather than a hydrogel dressing. Both hydrogel formulations maintained a hydrated material environment through their high swelling capacity, which may be relevant to topical wound management [49]. However, the present data do not permit attribution of the observed wound contraction differences to a specific mechanism. None of the animals presented clinical signs of infection during the experimental period. The comparison with silver sulfadiazine should therefore be regarded as a reference comparison rather than a direct equivalence trial.

No statistically significant differences in wound contraction were observed between the 1% EEP and propolis-free chitosan groups (*p* > 0.05). Thus, the present macroscopic data do not demonstrate an additional wound-contraction benefit attributable specifically to EEP at 144 h. Histologically, hair follicles and glandular structures were observed in several sections from the 1% EEP group. Because the histological assessment was descriptive and not quantitatively scored, these observations should be regarded as preliminary morphological findings rather than evidence that EEP preserved or regenerated cutaneous appendages. Because no EEP-free treatment group was included, the present design also does not allow separation of the effect of EEP alone from that of the chitosan matrix or demonstration of synergy between the two components.

These findings can be considered in the context of previous reports of propolis-containing biomaterials evaluated over longer periods [50]. However, the present study was limited to 144 h and did not include quantitative histological scoring or longitudinal assessment of appendage regeneration. Therefore, the current observations cannot establish when cutaneous appendages were preserved or regenerated, nor whether the observed differences persist during later proliferative and remodeling phases. Extended follow-up with quantitative histological and molecular endpoints will be necessary to address these questions.

The biological basis for this differential outcome between the 1% EEP and propolis-free chitosan groups warrants further investigation. Although the present study demonstrated sustained antioxidant activity and antimicrobial effects associated with the EEP-containing formulation, the chemical identity and relative abundance of individual propolis constituents were not determined. Therefore, specific molecular mechanisms cannot be established from the current data. Potential contributions of antioxidant and anti-inflammatory activities remain plausible hypotheses based on the broader propolis literature. The sustained antioxidant activity observed in the present study could potentially contribute to protection against oxidative stress; however, this interpretation was not directly investigated. Hair follicle stem cells and sebaceous gland progenitor cells are sensitive to oxidative stress [51]; therefore, an antioxidant contribution may be considered as a hypothesis rather than a demonstrated mechanism. Anti-inflammatory effects have also been reported for propolis and its constituents [52,53]. Nevertheless, the present study did not directly assess inflammatory signaling, cytokine production, or the molecular pathways responsible for these effects. The antimicrobial activity observed against *S. aureus* and *S. epidermidis* [43,44] could also contribute to the wound microenvironment, as these organisms are common early colonizers of burn wounds. Low-level bacterial colonization may sustain macrophage activation and pro-inflammatory signaling [54]; however, no overt clinical infection was observed in the present experiment. Accordingly, the present data do not permit attribution of the observed histological findings to specific flavonoids, individual compounds, or defined molecular pathways. Future studies combining HPLC-DAD, HPLC-MS, or related chromatographic phytochemical profiling with mechanistic assays would be required to establish composition–activity relationships and determine whether particular constituents contribute to the observed biological responses.

Collectively, the present data demonstrate that EEP incorporation produced measurable changes in the physicochemical, release, antioxidant, antimicrobial, and cell viability profiles of the chitosan hydrogel. The in vivo component provides preliminary evidence of early wound contraction and descriptive histological features at 144 h. However, the available data do not establish a specific mechanism for the observed tissue response, nor do they demonstrate enhanced regeneration or preservation of cutaneous appendages. Extended follow-up, quantitative histology, and molecular characterization will be required to determine whether the observed early differences translate into meaningful effects during later wound-healing phases.

The present study has some limitations that should be considered when interpreting the findings, including the absence of HPLC-based qualitative profiling of the propolis extract, the exploratory nature of the short-term in vivo and in vitro evaluations, static exposure conditions, the non-equivalent comparison with silver sulfadiazine, and the lack of tissue bioavailability, EEP-free controls, and quantitative histological assessment. Future work should therefore focus on higher-resolution molecular characterization of Chihuahua propolis using HPLC-DAD, HPLC-MS, or related chromatographic methods; dose–response in vivo studies of the 1%, 3%, and 5% EEP formulations with follow-up to 14, 21, and 28 days; assessment of skin permeation, deposition, and local exposure using models such as Franz cells; quantitative histological and immunohistochemical evaluation; and cytocompatibility studies under dynamic or controlled-release conditions using complementary assays, including Live/Dead staining, apoptosis/necrosis, cell-cycle analysis, and concentration–response/LC_50_ characterization of released flavonoids in NIH-3T3 cells. Expanded in vivo studies should also include EEP-free and vehicle controls. Together, these approaches will strengthen the evaluation of composition–activity relationships, dose–response behavior, and the translational safety of the formulations.

## 5. Conclusions

This study demonstrates the feasibility of a chitosan-based topical delivery platform incorporating ethanolic extract of Chihuahua propolis, characterized using parameters established in NOM-003-SAG/GAN-2017, for exploratory investigation in second-degree burn management. EEP incorporation provided sustained flavonoid release and radical-scavenging activity, enhanced swelling and structural persistence, and antimicrobial effects. Although EEP-containing hydrogels reduced NIH-3T3 viability below the 70% ISO 10993-5 criterion under static extraction conditions, no overt treatment-related clinical signs were observed during the short in vivo evaluation period. The antimicrobial findings revealed complementary diffusion- and contact-dependent effects, with the 5% EEP hydrogel reducing recoverable E. coli counts below the detection limit (<10^2^ CFU/mL). In the murine burn model, the 1% EEP hydrogel achieved greater wound contraction than silver sulfadiazine, although it did not differ significantly from the propolis-free chitosan hydrogel or untreated control. Qualitative histological assessment showed features compatible with early tissue repair, and cutaneous appendages were observed in several sections from the 1% EEP group. Because the histological assessment was descriptive and the follow-up was limited to 144 h, these observations should be considered preliminary and should not be interpreted as evidence of enhanced regeneration or preservation of cutaneous appendages. Collectively, these findings support further investigation of Chihuahua propolis-loaded chitosan hydrogels, including dose–response studies, dynamic cell-viability testing, quantitative histological assessment, extended follow-up, and comprehensive safety evaluation.

## Figures and Tables

**Table 1:** Physicochemical and biological parameters of the ethanolic propolis extract.

Evaluated Parameter	Results
Physical characterization (raw propolis)	
Color	Greenish-brown
Aroma	Pungent balsamic
Taste	Pungent
Consistency	Malleable
Antioxidant activity	
IC_50_ (µg/mL)	184.00 ± 9.70
Oxidation index (s)	31.33 ± 6.11
Bioactive compound quantification	
Total phenolic content (% gallic acid equivalents)	27.42 ± 2.53
Total flavonoid content (% quercetin equivalents)	9.23 ± 0.314
Hemolysis	
Erythrocyte hemolysis (%)	0.006 ± 0.0039
Brine shrimp lethality assay	
LC_50_ (µg/mL)	26.40 ± 4.75
Agar disc diffusion assay-inhibition zone (mm)	
*S. aureus*	14.33 ± 0.57
*E. coli* O157:H7	0 ± 0
*C. albicans*	21.66 ± 4.16
*S. epidermidis*	12.66 ± 0.57
Minimum inhibitory concentration (mg/mL)	
*S. aureus*	2.60
*E. coli* O157:H7	10.41
*C. albicans*	2.60
*S. epidermidis*	5.20

Values are expressed as mean ± standard deviation (SD) from three independent experiments (n = 3). MIC values represent the lowest concentration that inhibited visible metabolic activity (no resazurin color change) in at least two out of three independent replicates. A value of 0 in the inhibition zone indicates no inhibition detected under the conditions tested.

**Table 2:** Antimicrobial activity of chitosan–propolis hydrogels evaluated by agar disc diffusion assay.

Microorganism	Inhibition Zone Diameter (mm)			
	Chitosan	Chitosan + 1% Propolis (*w*/*w*)	Chitosan + 3% Propolis (*w*/*w*)	Chitosan + 5% Propolis (*w*/*w*)
*Staphylococcus aureus* (ATCC 25923)	0	10.7 ± 1.5	14.3 ± 3.2	17.0 ± 2.0
*Staphylococcus epidermidis* (ATCC 12228)	0	9.3 ± 1.5	12.3 ± 2.5	14.3 ± 2.9
*Escherichia coli* O157:H7 (ATCC 43895)	0	0	0	0
*Candida albicans* (ATCC 14053)	0	11.0 ± 2.8	16.0 ± 1.4	20.0 ± 4.2

Values expressed as mean ± SD (n = 3). Results reported as inhibition zone diameter (mm). 0 indicates no inhibition zone detected under the conditions tested.

## Data Availability

The original contributions presented in this study are included in the article. Further inquiries can be directed to the corresponding author.

## Abbreviations

The following abbreviations are used in this manuscript:

ATR	Attenuated Total Reflectance
BHI	Brain Heart Infusion
CAPE	Caffeic Acid Phenethyl Ester
CFU	Colony-Forming Unit
CO_2_	Carbon Dioxide
DMEM	Dulbecco’s Modified Eagle’s Medium
DMSO	Dimethyl Sulfoxide
DPPH	2,2-Diphenyl-1-picrylhydrazyl
DSC	Differential Scanning Calorimetry
EEP	Ethanolic Extract of Propolis
FBS	Fetal Bovine Serum
FTIR-ATR	Fourier Transform Infrared Spectroscopy–Attenuated Total Reflectance
H&E	Hematoxylin and Eosin
ISO	International Organization for Standardization
LC_50_	Median Lethal Concentration
MIC	Minimum Inhibitory Concentration
NF-κB	Nuclear Factor Kappa B
NIH-3T3	Mouse Embryonic Fibroblast Cell Line NIH-3T3
PBS	Phosphate-Buffered Saline
QE	Quercetin Equivalents
ROS	Reactive Oxygen Species
SEM	Scanning Electron Microscopy
SD	Standard Deviation
TGA	Thermogravimetric Analysis
UV	Ultraviolet
UV-Vis	Ultraviolet–Visible Spectrophotometry
*w*/*w*	Weight per Weight
